# Winning Combination of Cu and Fe Oxide Clusters with an Alumina Support for Low-Temperature Catalytic Oxidation of Volatile Organic Compounds

**DOI:** 10.1021/acsami.3c02705

**Published:** 2023-06-02

**Authors:** Tadej Žumbar, Iztok Arčon, Petar Djinović, Giuliana Aquilanti, Gregor Žerjav, Albin Pintar, Alenka Ristić, Goran Dražić, Janez Volavšek, Gregor Mali, Margarita Popova, Nataša Zabukovec Logar, Nataša Novak Tušar

**Affiliations:** † National Institute of Chemistry, Hajdrihova 19, SI-1000 Ljubljana, Slovenia; ‡ University of Nova Gorica, Vipavska 13, SI-5000 Nova Gorica, Slovenia; § Elettra-Sincrotrone Trieste S.C.p.A., Strada Statale 14 - km 163,5 in AREA Science Park, 34149 Basovizza, Trieste, Italy; ∥ Institute of Organic Chemistry with Centre of Phytochemistry, Bulgarian Academy of Sciences, Acad. G. Bonchev Str., Bl. 9, 1113 Sofia, Bulgaria

**Keywords:** Iron oxide clusters, copper oxide clusters, alumina support, synergistic effect, low-temperature total catalytic oxidation, toluene

## Abstract

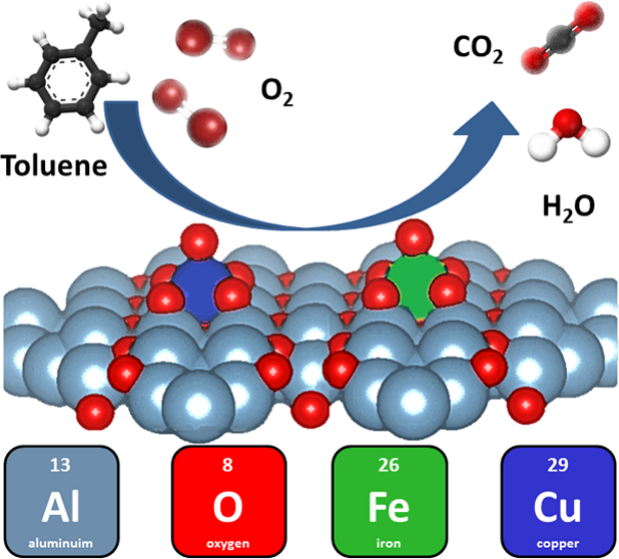

A γ-alumina support functionalized with transition metals is one of the most widely used industrial catalysts for the total oxidation of volatile organic compounds (VOCs) as air pollutants at higher temperatures (280–450 °C). By rational design of a bimetal CuFe-γ-alumina catalyst, synthesized from a dawsonite alumina precursor, the activity in total oxidation of toluene as a model VOC at a lower temperature (200–380 °C) is achieved. A fundamental understanding of the catalyst and the reaction mechanism is elucidated by advanced microscopic and spectroscopic characterizations as well as by temperature-programmed surface techniques. The nature of the metal–support bonding and the optimal abundance between Cu–O–Al and Fe–O–Al species in the catalysts leads to synergistic catalytic activity promoted by small amounts of iron (Fe/Al = 0.005). The change in the metal oxide–cluster alumina interface is related to the nature of the surfaces to which the Cu atoms attach. In the most active catalyst, the CuO_6_ octahedra are attached to 4 Al atoms, while in the less active catalyst, they are attached to only 3 Al atoms. The oxidation of toluene occurs via the Langmuir–Hinshelwood mechanism. The presented material introduces a prospective family of low-cost and scalable oxidation catalysts with superior efficiency at lower temperatures.

## 1 Introduction

One of the main concerns in environmental protection is air pollution and the means to control or reduce it. Among different pollutants with subsequent harmful effects, such as carbon monoxide, ammonia, NO*_x_*, SO*_x_*, and particulate matter, are also volatile organic compounds (VOCs).^1^ Depending on the source of pollution with such organic matter, different techniques are applied, among which is also the catalytic oxidation of the exhaust fumes (flue gas).^2,3^ There are two main groups of catalysts used for this operation:^4^ noble metals (working at 150–280 °C) and transition metal based (working at 280–450 °C). Noble metals require lower operating temperatures and, therefore, usually lower operation costs.^5^ Nonetheless, their drawbacks such as price, availability, geographical distribution, and tendency to self-poisoning give focus to the catalysts based on transition metals. Even more, a synergistic effect was observed in some cases over bimetallic transition metal catalysts that enhanced their activity at lower temperatures in comparison to monometallic ones,^6,7^ which increased the interest in the research topic and tendency to replace the established noble metal catalysts.

Copper oxide on different supports is recognized as one of the most prosperous catalysts for the total catalytic oxidation of volatile organic compounds. Studies have shown that not only the active phase but also the type and the structure of the support and possible active phase–support interactions that form as a consequence even improve the performance of such catalysts.^8,9^

Our group previously showed that a bimetallic Cu–Fe dispersed over amorphous silica enables both homogeneous dispersion of the active sites and good stability of the active phase. Furthermore, the bimetallic Cu–Fe silica-supported catalyst exhibits promoted catalytic oxidation of toluene as a model VOC,^10^ as the specific copper/iron = 11 molar ratio led to a most intense cooperative redox effect between CuO nanocrystals and dispersed Cu-oxo-Fe clusters.

Two reaction mechanisms are generally used to explain the total catalytic toluene oxidation.^11,12^ In the redox mechanism (Mars–van Krevelen mechanism), the adsorbed toluene is oxidized with O_latt_ in the redox-active component of the catalyst, which results in the reduction of metal oxides. The reduced metal oxides are oxidized again by O_2_ from the gas phase in order to complete the cyclic redox process. The oxygen vacancy is generated when the O_latt_ is consumed during the redox reaction.^13,14^ As a result, oxygen activation, dissociation, and the replenishment of O_latt_ play a key role in toluene oxidation. Alternatively, toluene can be oxidized without the involvement of lattice oxygen species (Langmuir–Hinshelwood mechanism), namely, by different surface adsorbed oxygen species (such as superoxide radicals or anions), without change in the oxidation state of the active sites.^15−17^ Temperature-programmed and isothermal transient reduction and oxidation experiments can be applied to analyze the structure- and composition-dependent redox properties of the materials and their relevance for the catalytic activity.

In this work, we investigated the structure–property–activity relationship of the bimetal CuFe-γ-alumina catalyst for the gas-phase total oxidation of toluene as a model VOC at a lower temperature (200–380 °C). The driving force for this study previously gained knowledge on the promising catalytic behavior of the Cu–Fe containing mesoporous silica at a copper/iron molar ratio of 11.^10^ The study focused on Cu–Fe containing alumina-supported catalysts and their behavior in the mentioned reaction^18^ and alumina as an affordable material. The latter study showed that the alumina support characteristics play an important role in the performance of such catalysts. Alumina can be produced from various aluminum materials, among which the dehydration of different aluminum hydroxides is the most common and economically feasible one.^19^ A dawsonite alumina precursor was used for this study.

## 2 Experimental Section

### 2.1 Synthesis

For this work, Cu–Fe-functionalized alumina supports synthesized from an ammonium dawsonite (NH_4_AlCO_3_(OH)_2_) precursor with different Fe loadings (from Fe/Al = 0.005 to Fe/Al = 0.05) and constant Cu loading (8 wt %) were prepared using a two-step synthesis approach. Dawsonites are compounds with a chemical composition AMCO_3_(OH)_2_; A represents a cation (Na^+^, K^+^, NH_4_^+^, Mg^2+^, Ca^2+^, Ba^2+^), and M is usually a trivalent metal ion such as Al^3+^.^20,21^ Alumina derived from ammonium dawsonites tend to possess high specific surface area and chemical purity, as all constituents of this dawsonite type are removed in the gas phase during thermal treatment.^20,22^ Ammonium aluminum carbonate hydroxide was synthesized with ammonium carbonate solution (2 M, Honeywell Fluka, Puriss.) and aluminum chloride solution (2 M, Alfa Aesar, 99%), combined in a molar ratio of 4:1 in favor of the basic compound. The synthesis procedure was based on the data in^23,24^ with some minor modifications like using chloride instead of nitrate and altering the molar ratio between the compounds. Iron was introduced during the neutralization reaction via 0.146 M iron(III) chloride hexahydrate (VWR Chemicals, 99.4%) solution in a quantity that corresponds to the desired Fe/Al molar ratio in the calcined aluminum oxide. The ingredients were mixed using a peristaltic pump and pH control, which was set to 8. After that, the suspension was heated to 85 °C and aged for 3 h under vigorous stirring. The product was filtered and washed with deionized water. To prevent an undesirable boehmite formation,^23^ a 30:1 water against Al_2_O_3_ mass ratio was used. The washed dawsonite was then dried for 24 h at 105 °C. The material was then calcined to transform the structure of dawsonite into alumina. Calcination was performed for 2 h at two chosen temperatures, 500 and 1000 °C in the presence of air. Copper was deposited using the incipient wetness impregnation method, using Cu(NO_3_)_2_·3H_2_O (Merck, EMSURE) solution in an amount that corresponds to 8 wt % of CuO in the catalyst. Prior to use, the impregnated materials were dried and calcined at 500 °C for 1 h. The samples were denoted as the D-iron/aluminum ratio-temperature of calcination-copper presence. For example, the sample D-0.01Fe-500-Cu has a 0.01 Fe/Al ratio, was calcined at 500 °C, and impregnated with copper. Spent catalysts are denoted by R. Two commercially used catalysts were acquired for a comparison of catalytic activity: Catalyst Type 50B from Johnson Matthey (JM-0.5Pd, 0.5 wt % Pd on alumina) and HNC-30 from Hulteberg, denoted as Cu/Mn–Al_2_O_3_, containing a combination of copper (3.8 wt % as CuO) and manganese (10.8 wt % as MnO) on an alumina support.

### 2.2 Characterization

X-ray diffraction measurements were done on a PANalytical X’Pert PRO MPD with Cu Kα_1_ radiation of λ = 1.5406 Å, at ambient conditions between 5 and 80° 2Θ with a step of 0.034° and 100 s per step. Diffractograms were analyzed by X’Pert software.

Thermal and differential thermal analyses (TG/DTG) were performed on a Q5000IR analyzer from TA Instruments. The analysis was performed from room temperature to 950 °C with a heating ramp of 10° min^–1^ in an air flow stream of 25 mL min^–1^.

Scanning electron microscopy (SEM) was performed using a Zeiss Supra TM 35 VP microscope.

Elemental analysis was performed on a BRUKER AXS S8-TIGER X-ray fluorescence (XRF) spectrometer with a 1 kW rhodium tube and PET, XS-55, XS-Ge-C, and LiF200 analyzer crystals with vacuum.

The N_2_ physisorption was conducted with a Micromeritics Tristar 3000 volumetric adsorption analyzer. Prior to the analysis, degassing at 200 °C for 12 h was performed. The Brunauer–Emmett–Teller (BET)^25^ specific surface area was determined from adsorption data in the relative pressure range from 0.05 to 0.30. The total pore volume was estimated at a relative pressure of 0.98. Pore size distributions were calculated based on the adsorption BJH logarithm,^25^ and pore sizes were determined at a maximum of these distributions.

The concentration of acid sites and their relative strength were determined using a Perkin Elmer TGA Pyris 1 thermogravimetric analyzer (TGA) coupled to a thermal analysis gas station (TAGS) and pyridine as a probe molecule. The sample was preheated to 500 °C for 15 min with a heating ramp of 10 °C min^–1^ in an air stream of 50 mL min^–1^. The sample was cooled to 120 °C with the same ramp and held at that temperature for 10 min. The gas was switched from air to nitrogen (30 mL min^–1^), and pyridine was introduced until a steady sample weight was achieved. Afterward, the sample remained at 120 °C for 120 min to degas the weakly bound pyridine. The heating step to 500 °C with the ramp of 20 °C min^–1^ followed and the cooling to room temperature just after. The concentration of acid sites was calculated based on the weight difference of the sample before and after the saturation of pyridine and was presented as the total number of moles of pyridine per gram or square meter of the sample. The strength of acid sites was estimated based on the pyridine bonding strength as a derivative of the temperature-programmed desorption curve, taking into account that stronger acid sites release pyridine at higher temperatures. Results were displayed as temperature-programmed desorption curves with peak maxima indicating the pyridine desorption temperature.

Ultraviolet–visible (UV–vis) diffuse reflectance (DR) spectra were recorded on a Perkin Elmer Lambda 35 spectrophotometer equipped with a Praying Mantis accessory. The background was recorded with a Spectralon reference. Samples were scanned in the spectral range between 200 and 900 nm, with a slit set to 2 nm and a scanning speed of 240 nm min^–1^.

Solid-state ^27^Al magic-angle spinning (MAS) NMR spectra were recorded on a 600 MHz Varian NMR instrument, operating at an ^27^Al Larmor frequency of 156.178 MHz. The sample rotation frequency was 20 kHz, duration of the excitation pulse was 1 μs, repetition delay between consecutive scans was 0.5 s, and the number of scans for each spectrum was 1000. In all spectra, the frequency axis in ppm is reported relative to the signal of Al nuclei within 1 M Al(NO_3_)_3_ solution.

Cu and Fe K-edge X-ray absorption spectra (XAS) of Cu- and Fe-functionalized alumina catalysts were recorded in transmission or fluorescence detection mode at the X-ray absorption fine structure (XAFS) beamline of the ELETTRA synchrotron radiation facility in Trieste, Italy. The analysis of X-ray absorption near edge structure (XANES) and extended-XAFS (EXAFS) spectra was performed with the DEMETER (IFEFFIT) program package^26^ in combination with the FEFF6 program code^27^ for ab initio calculation of photoelectron scattering paths.

Morphology characteristics, the distribution of copper and iron, and their structural correlation with the alumina support were investigated by transmission electron microscopy (TEM) and scanning transmission electron microscopy (STEM). The analysis was performed on a Cs probe-corrected STEM JEOL ARM 200 CF with a cold-FEG cathode. The latter was equipped with a dual-EELS system Quantum ER from the Gatan and Centurio energy-dispersive X-ray spectroscopy (EDXS) system with a 100 mm^2^ silicon drift detector (SDD). For TEM studies, a drop of an ethanol diluted sample suspension was placed on a lacey-carbon-coated nickel grid and dried at room temperature. Two observation techniques were used in the STEM mode, high-angle annular dark-field (HAADF) imaging and bright-field (BF) imaging.

An AutoChem II 2920 apparatus (Micromeritics) was used for H_2_-TPR analysis. A powdered catalyst sample (≈50 mg) was positioned on a quartz wool flock inside a U-shaped quartz tube and pretreated in a 5% O_2_/He stream (25 mL min^–1^) for 10 min at 400 °C. After cooling to 50 °C, the gas atmosphere was switched to Ar for 10 min and afterward to 5% H_2_/Ar (25 mL min^–1^). During analysis, the sample temperature was increased to 700 °C with a heating ramp of 10 °C min^–1^. The reduction profiles were recorded using a thermal conductivity detector (TCD). A liquid isopropanol/LN_2_ cold trap (*T* ≈ −80 °C) was used in order to condense water and eliminate its contribution to the recorded H_2_-TPR profiles.

Temperature-programmed desorption of oxygen (TPD-O_2_) was performed using a Micromeritics AutoChem II 2920 apparatus connected to a ThermoStar mass spectrometer (Pfeiffer vacuum). The samples (≈100 mg) were pretreated at 400 °C with synthetic air for 10 min and cooled down to room temperature. Then, the carrier gas stream was switched to He for 15 min (purging), followed by the temperature-programmed increase to 700 °C with a ramp of 10 °C min^–1^. Oxygen, carbon dioxide, and water were detected during desorption using a ThermoStar mass spectrometer. The amount of desorbed oxygen during TPD-O_2_ runs was determined by means of pulsing the known oxygen amount to the mass spectrometer prior to the analysis.

The temperature-programmed toluene oxidation reaction (TPD/R-toluene) was carried out using the Micromeritics II 2920 AutoChem apparatus. Prior to experiments, the catalysts (≈100 mg) were pretreated at 400 °C for 30 min and cooled to 50 °C in 5% O_2_/He flow (25 mL min^–1^). The sample was then flushed in Ar at 25 mL min^–1^ for 20 min and saturated with a series of 20 toluene pulses generated in a vapor generator, which was heated to 81 °C (30% of toluene in the injection loop). For the analysis, the sample was first flushed with Ar (25 mL min^–1^) for 15 min, and then the temperature was raised to 600 °C with a heating ramp of 10 °C min^–1^. Desorption of toluene (*m*/*z* = 91) and O_2_ (*m*/*z* = 32), as well as the formation of reaction products (H_2_O, *m*/*z* = 18; CO, *m*/*z* = 29; CO_2_, *m*/*z* = 44), were analyzed using a mass spectrometer (Pfeiffer vacuum, model ThermoStar).

The pulsed toluene oxidation reaction was carried out in the presence of a D-500-Cu catalyst using the Micromeritics II 2920 AutoChem apparatus. Prior to experiments, the catalyst (≈20–50 mg) was pretreated at 400 °C for 30 min and cooled to 380 °C in 5% O_2_/He flow (25 mL min^–1^), followed by purging in Ar (25 mL min^–1^) for 10 min. The reaction temperature of 380 °C was chosen based on the results of the catalytic activity of the catalysts, discussed later in the text. The reaction was then carried out either in an inert (Ar at 25 mL min^–1^) or air (25 mL min^–1^) atmosphere using a series of 20 toluene pulses generated in a vapor generator, which was heated to 81 °C (30% of toluene in the injection loop). Conversion of toluene (*m*/*z* = 91) as well as the formation of H_2_O (*m*/*z* = 18), CO (*m*/*z* = 29), and CO_2_ (*m*/*z* = 44) was analyzed using a mass spectrometer (Pfeiffer Vacuum, model ThermoStar).

### 2.3 Catalytic Tests

Catalytic tests were performed in a stainless-steel fixed-bed reactor (10 mm I.D.), previously proved inert. The temperature was controlled with a probe inserted in the packing area and connected to a PID regulator to ensure a stable and controllable operation of the reactor setup (which is presented in Figure S1) The outlet of the reactor was connected to an Agilent micro GC chromatograph equipped with integrated PPU and 5CB columns and a TCD detector. The outlet line of the reactor was heated to prevent unwanted condensation. For each test, 400 mg of a catalyst with a particle size between 0.2 and 0.8 mm was placed in a packed bed consisting of quartz wool and a known amount of inert tabular alumina grains (2–3 mm). Pretreatment of the catalyst was done prior to each test in situ at 400 °C for 1 h in a pure nitrogen stream. To study the catalyst activity, the reactor was cooled to 200 °C, and the gas reactants were introduced. The gas stream consisted of 100 mL min^–1^ synthetic air (Messer) passing through the toluene solution at 0 °C (partial pressure of toluene 0.9 kPa). The concentration of toluene in gas vapor was around 1%. The temperature was raised stepwise to 480 °C and analysis of the outlet gas stream was performed in 20 °C intervals. Conversion of toluene was calculated as *X*_T_ = (*c*_T,IN_ – *c*_T,OUT_)/*c*_0_ × 100 (%), where *c*_T,IN_ represents the inlet toluene concentration, *c*_T,OUT_ represents the measured outlet concentration of toluene at a certain temperature, and *X*_T_ is the conversion at this temperature. The only detected reaction products were CO_2_ and water. The catalytic stability of the samples was tested in a similar manner: After pretreatment, the reactor was cooled down to a temperature at which 50% of the conversion was reached (*T*_50_) during the activity tests for the best working catalyst. The time-on-stream (TOS) test was performed isothermally for a period of 23 h at 380 °C.

Carbon content on spent catalysts was determined by means of CHN elemental analysis using a Perkin Elmer 2400 Series II CHNS/O elemental analyzer.

## 3 Results and Discussion

### 3.1 Catalyst Description

Cu–Fe functionalized alumina supports synthesized from an ammonium dawsonite precursor with different Fe loadings (from Fe/Al = 0.005 to Fe/Al = 0.05) and constant Cu loading (8 wt %) were prepared using a two-step synthesis approach. In the first step, Fe alumina samples were prepared via direct synthesis and calcined at two chosen temperatures, 500 and 1000 °C in the presence of air. In the second step, Cu was added via impregnation following solid-state thermal conversion (chapter 2.1. and Figure S2). Copper-only loaded samples (8 wt % of Cu) were also prepared.

**Figure 1:**
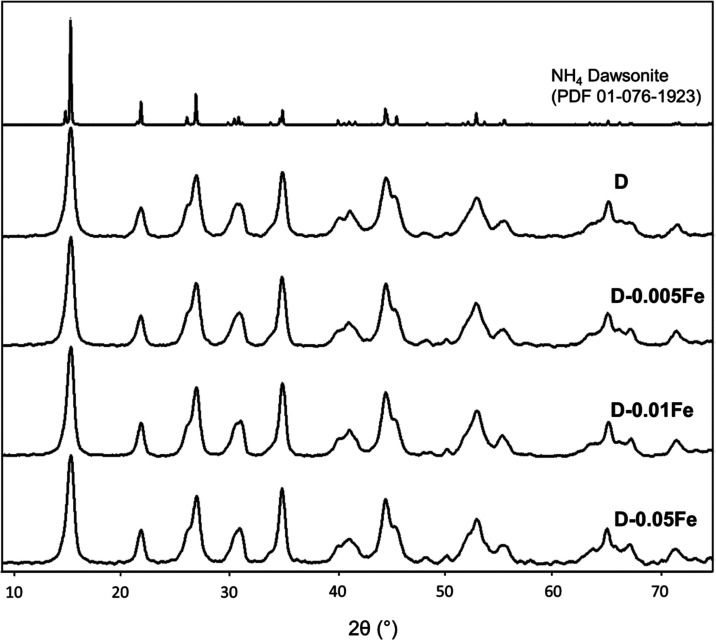
XRD patterns of prepared dawsonites with increasing iron to aluminum molar ratios and the dawsonite reference pattern PDF 01-076-1923.

X-ray diffraction analysis of the samples confirmed the presence of the dawsonite structure in the synthesized samples, regardless of the iron content (Figure 1) and with a total absence of the boehmite phase (AlO[OH]), which can be a consequence of the described synthesis procedure.^23^

**Figure 2:**
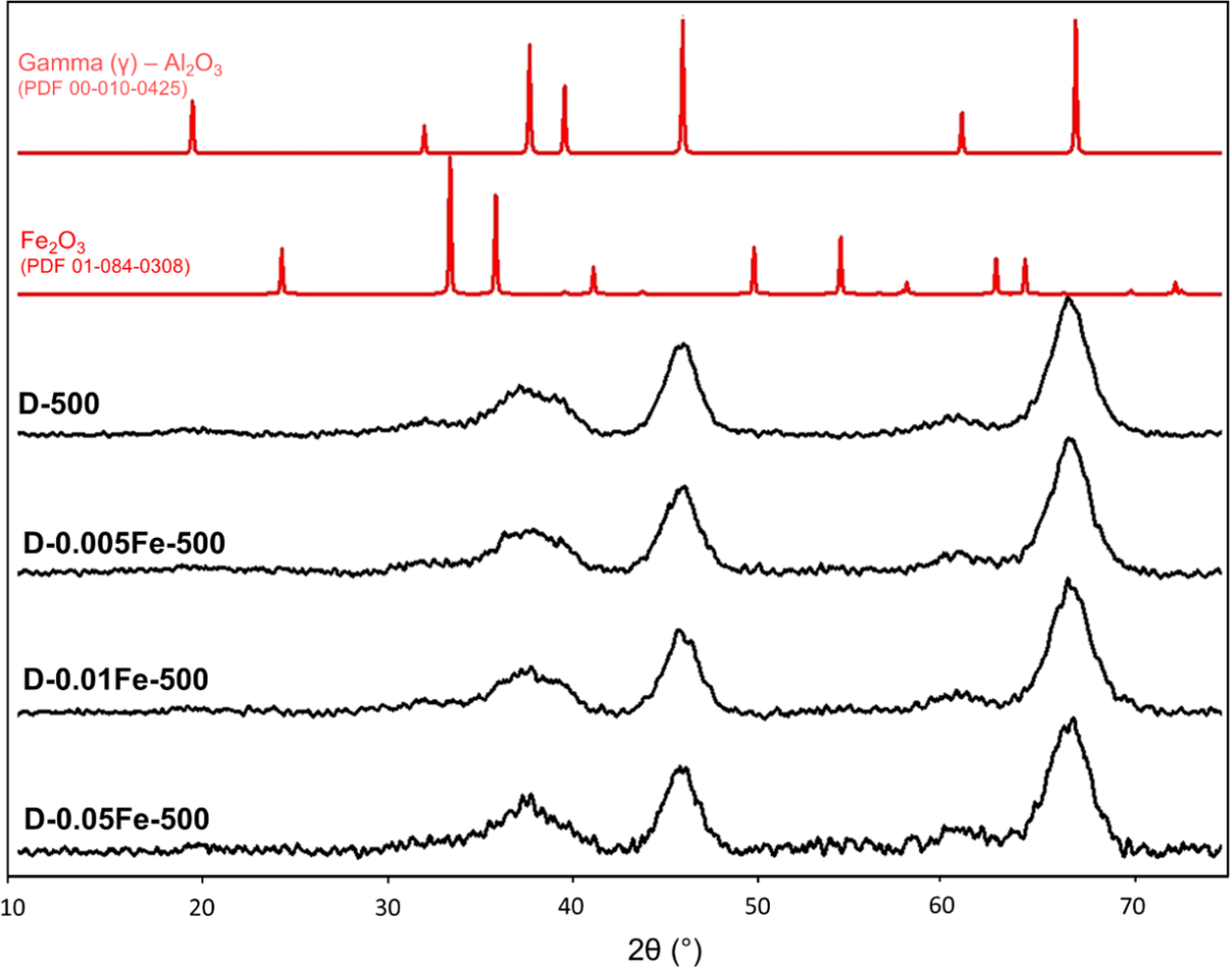
XRD patterns of calcined dawsonites at 500 °C with different iron contents as well as γ-alumina and Fe_2_O_3_ reference patterns (PDF 00-010-0425 and PDF 01-084-0308).

The dawsonite is completely transformed to γ-alumina after calcination at 500 °C (Figure 2) and to the mixture of θ-alumina and δ-alumina after calcination at 1000 °C (Figure S3), and no iron oxide particles were detected with XRD. When the iron content is increased, there is potential that iron oxide or other iron-containing structures/aggregates form during synthesis. However, their presence can only be confirmed by XRD when their particle size exceeds 4 nm (cca. 10 times unit cell).

**Figure 3:**
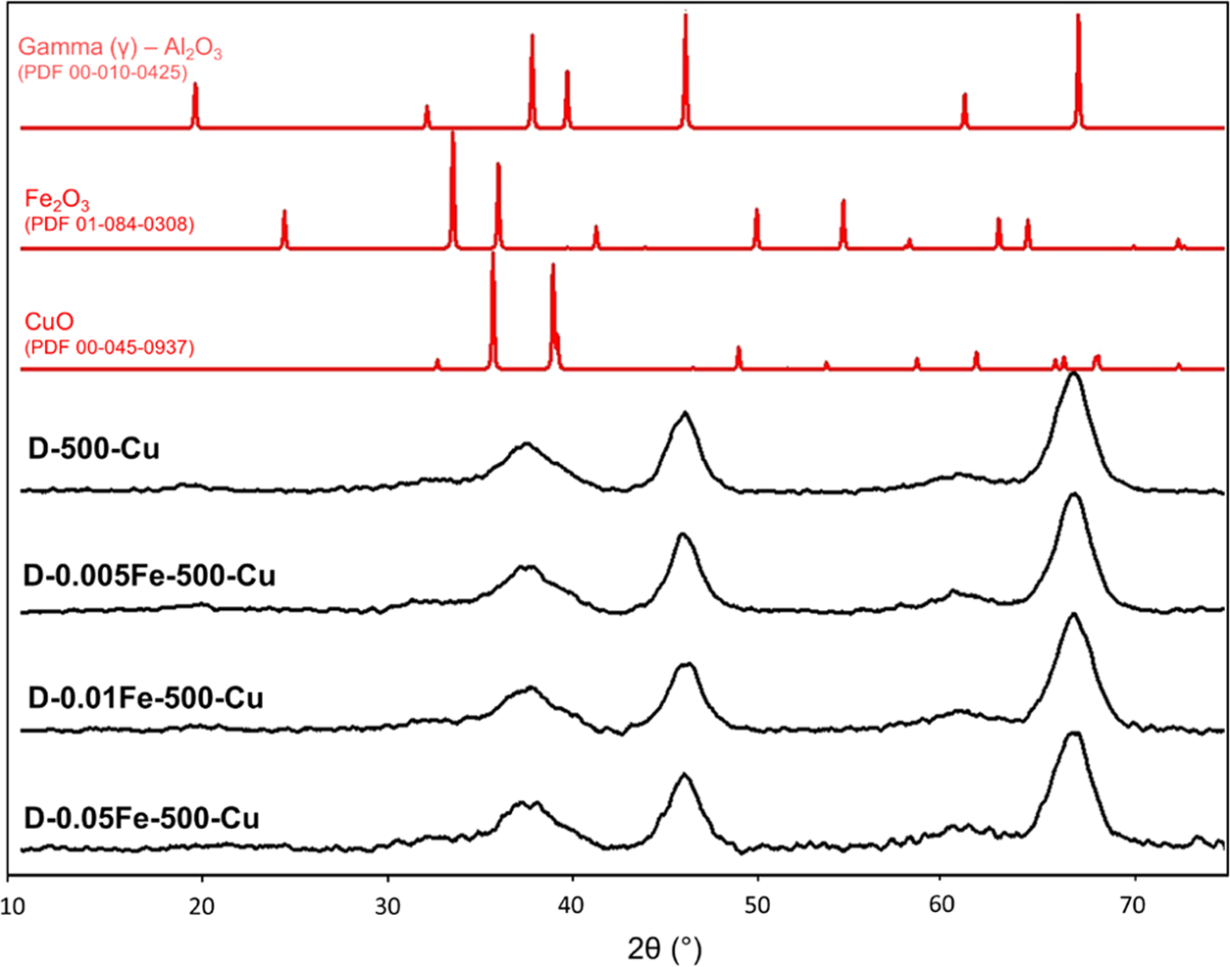
XRD patterns of calcined dawsonites at 500 °C with different iron contents and a constant amount of Cu together with reference patterns of copper (II) oxide, iron (III) oxide, and γ-alumina (PDF 00-045-0937, PDF 01-084-0308, and PDF 00-010-0425).

After impregnation of the γ-alumina supports with copper, the XRD patterns do not show any structural changes. No crystalline copper oxide particles were observed, independently of the iron concentration in the alumina support structure. The presence of small crystalline copper oxide nanoparticles or the amorphous CuO phase is again not excluded, but very small Cu particles (below 5 nm-cca. 10 times unit cell) or amorphous CuO phases cannot be confirmed by XRD analysis (Figures 3 and S3).

Elemental analysis and textural properties of the prepared catalysts (surface area, pore volume, acid sites) are presented in Table 1. The specific surface area of the catalysts ranges from 411 m^2^ g^–1^ for the bare alumina support (D-500) to 230 m^2^ g^–1^ for the Cu–Fe alumina sample with the highest amount of Cu and Fe. No distinct changes can be observed in the morphology of the samples, regardless of the iron content (Figure S4). After copper impregnation onto the iron-containing alumina supports calcined at 500 °C, the morphology remains intact. The morphology (that is visible in the range of SEM analysis) also remained preserved in the case of (Fe-doped) alumina supports being calcined at 1000 °C.

**Table 1:** Textural Properties, Acid Site Concentration (a.s.c.), Specific Surface Area (*S*_BET_), Pore Volume (*V*_P_), Pore Diameter (*d*_P_), and Chemical Composition Determined by XRF Analysis and Presented as Fe/Al and Cu/Al Molar Ratios

		N_2_ physisorption			chemical composition [molar ratio]	
sample	a.s.c. [mmol g^–1^]	*S*_BET_ [m^2^ g^–1^]	*V*_P_ [cm^3^ g^–1^]	*d*_P_ [nm]	Fe/Al	Cu/Al
D-500	0.419	411	1.28	5, 32.5, 42.5		
D-500-Cu	0.264	229	0.49	6, 40		0.067
D-1000	0.111	141	0.81	39		
D-1000-Cu	0.117	122	0.68	32		0.061
D-0.005Fe-500	0.308	374	1.42	5, 29, 34, 43	0.005	
D-0.005Fe-500-Cu	0.256	256	0.80	7, 29	0.007	0.054
D-0.005Fe-1000	0.119	148	0.78	31	0.005	
D-0.005Fe-1000-Cu	0.115	122	0.64	38	0.007	0.053
D-0.01Fe-500	0.391	391	1.45	5 in 30	0.010	
D-0.01Fe-500-Cu	0.351	235	0.26	4	0.015	0.063
D-0.05Fe-500	0.418	357	1.42	5, 31	0.048	
D-0.05Fe-500-Cu	0.375	230	0.28	4	0.056	0.069

The acidity of the alumina support enables electronic interactions and attraction between the electron-rich aromatic ring of toluene and electron-deficient acid sites, thus enabling stronger adsorption.

The presence of iron does not influence the acidity of materials, as they contain similar acid site concentration (a.s.c.) compared to pure transition alumina. With the introduction of copper, an obvious trend of increasing a.s.c. with an increased iron content (increased Fe/Al molar ratio) starts to appear, although the total a.s.c. is reduced if compared to iron-containing alumina (Table 1). This is probably due to the reduced specific surface area occupied by the copper-containing species. The increase of a.s.c. with an increased Fe/Al ratio could be explained by more copper being incorporated or bound that leads to new Lewis acid sites, likely due to the interaction with Fe.

### 3.2 Spectroscopic and Microscopic Characterization of Catalysts

#### 3.2.1 UV–VIS Diffuse Reflectance Spectroscopy

**Figure 4:**
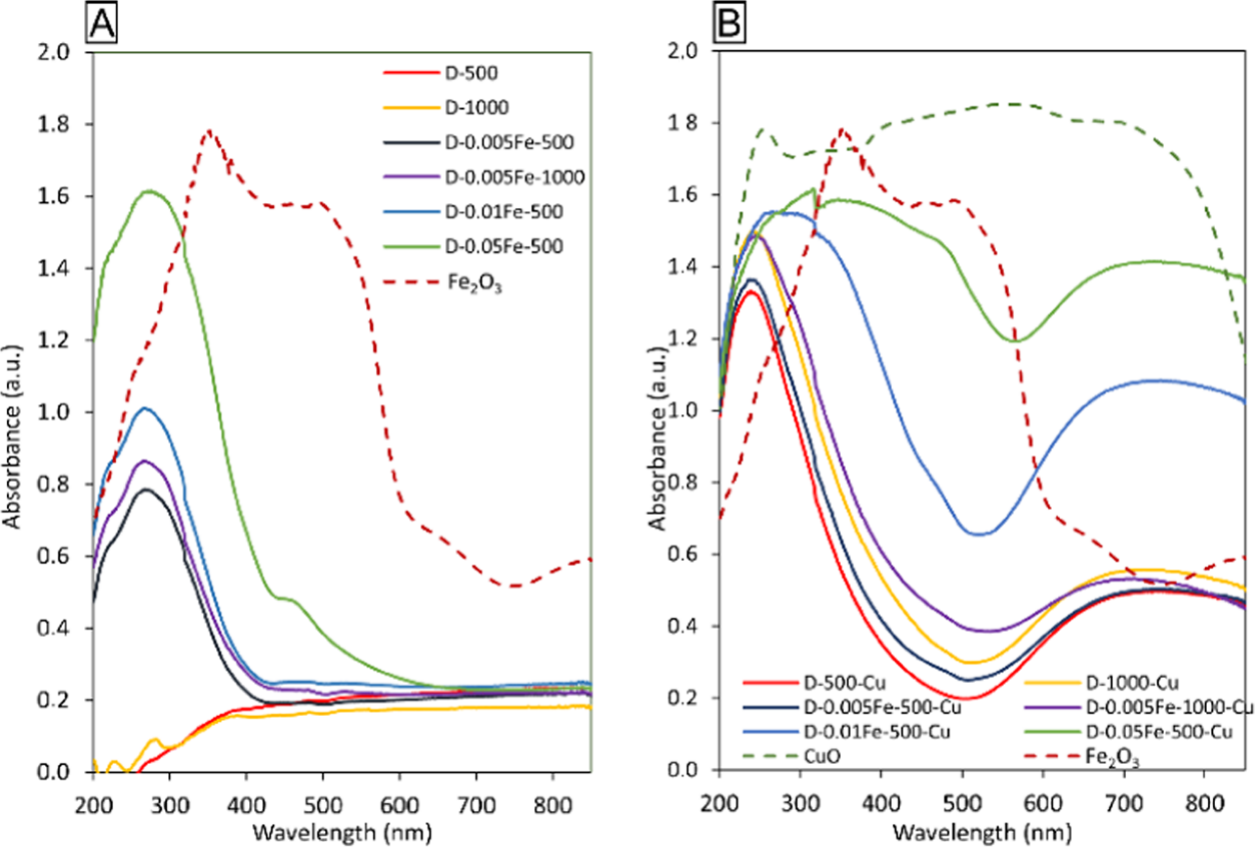
UV–vis-DR spectra of samples prepared without copper (A) and with copper (B). It has to be noted that the size of the particles in the bulk samples is not the same as in the studied samples. So the differences in UV–vis spectra between the bulk oxides and the studied samples can be observed.

The UV–vis-DR spectra, which were recorded to understand the coordination environment of Cu and Fe species in the samples, were visually very different (Figure 4). The UV–vis spectrum of the bulk α-Fe_2_O_3_ shows four absorption bands in the near UV, visible, and near IR regions: 350, 510, 650, and 850 nm, which can be assigned to metal–ligand charge-transfer, double excitation processes, and ligand field transitions, respectively.^28^ The UV–vis spectrum of the CuO bulk is characterized by the presence of a band at 250 nm, which can be attributed to the ligand–metal charge transfer from O^2–^ to Cu^2+^ in octahedral coordination, and by a very broad band between 380 and 850 nm, which can be assigned to the contributions of the d–d transitions.^29^ The UV–vis-DR spectra of iron-containing zeolites and mesostructured silicas, as the closest well-studied analogues of investigated materials, are characterized by intense Fe^3+^ → O charge-transfer bands, the position of which provides information on the iron coordination.^30,31^ Isolated Fe^3+^ ions give rise to bands below 300 nm, and signals of dinuclear bridged Fe(III)-oxo clusters appear between 300 and 400 nm, whereas bands at >400 nm can indicate the presence of oligomeric Fe*_x_*O*_y_* species.^32^ Bands observed at wavelengths >500 nm originate from agglomerated iron oxide particles.^33^

The incorporation of iron into Al_2_O_3_ during synthesis is clearly evident in Figure 4a. The spectra of the samples D-0.005Fe-500, D-0.005Fe-1000, and D-0.01Fe-500 reveal bands at 223 and 272 nm and a shoulder at 346 nm. The first two bands are assigned to the charge transfer between the iron and oxygen atoms of Fe–O–Al in the structure, which indicates the presence of tetrahedrally and octahedrally coordinated Fe^3+^ species, respectively. The shoulder at 346 nm is assigned to octahedral Fe^3+^ in dinuclear Fe-bridged complexes.^34^ It can be observed that the intensity increases with the increased amount of iron in the samples. The highest amount of iron is present in the sample D-0.05Fe-500, showing bands at 221 and 280 nm, and the shoulder at 367 nm with an additional band at around 466 nm. The latter band can be assigned to oligomeric Fe-oxo clusters.^32^ A weak band at 520 nm is observed in the samples D-0.005Fe-1000 and D-0.01Fe-500 due to the presence of Fe_2_O_3_ nanoparticles, while this band in the D-0.05Fe-500 sample shows a higher intensity, revealing that the iron amount and not the calcination temperature is the main driver for iron oxide nucleation.

UV–vis-DR spectra (Figure 4b) of copper and iron-containing samples reveal a Cu^2+^ → O charge-transfer (CT) band at 250 nm, which is usually assigned to isolated Cu^2+^ ions.^33^ Bands between 350 and 500 nm observed in all samples can be assigned to various oligomeric (CuO*_x_*) copper-oxo clusters.^35^ Furthermore, the band between 500 and 850 nm corresponds to the d–d transition of Cu^2+^ ions in the pseudo-octahedral ligand oxygen environment, indicating the formation of Cu–O–Al.^36^ These results are in good agreement with the EXAFS results. The UV–vis-DR spectra of D-0.01Fe-500-Cu and D-0.05Fe-500-Cu samples are different from the other copper-containing samples, namely, more intense bands are observed in the ranges between 400 and 500 nm and between 500 and 850 nm, indicating the presence of a higher content of various oligomeric CuO*_x_* clusters and Cu–O–Al bridged complexes,^35^ respectively. The UV–vis-DR spectrum of the D-0.05Fe-500-Cu sample shows a broad, intense band at 480 nm, which is present in all iron- and copper-containing samples but not in the samples containing only Cu, and it may correspond to a combined contribution of oligomeric Cu-oxo and Fe-oxo clusters.^37^ The intensity of this band and, consequently, the amount of both clusters decreases with a decreasing amount of iron.

#### 3.2.2 ^27^Al MAS NMR

**Figure 5:**
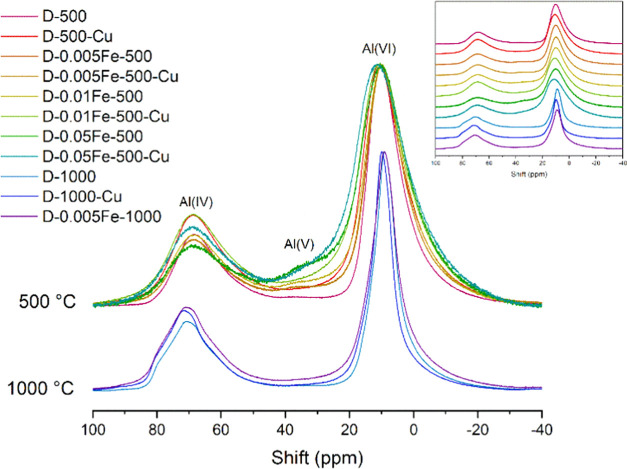
^27^Al MAS NMR spectra of samples calcined at 500 (γ-alumina) and 1000 °C (mixture of θ-alumina and δ-alumina). For better clarity, an inset with stacked spectra is added. Note that spectra of D-0.005Fe-500 and D-0.005Fe-500-Cu samples are almost identical. This is more clearly presented in the Supporting Material, where detailed fits of ^27^Al MAS NMR spectra of both samples are shown (Figure S8).

^27^Al MAS NMR spectra of the D-1000 originating samples exhibit strong signals of tetra- (IV) and octahedrally (VI) coordinated aluminum atoms at about 70 and 10 ppm, respectively. In addition to such signals, the spectra of the D-500 series also show a weak signal of five (V) coordinated aluminum atoms, confirming the presence of a small fraction of the amorphous material with AlO_5_ groups in these samples. The ratio between Al(IV) and Al(VI) is very similar in all D-1000 and D-500 samples. The major difference is that the spectra of the D-1000 series of samples have narrower and better-defined quadrupole lines, which suggests that these materials have more ordered structures than the materials of the D-500 series (Figures 2 and S3). Comparison of the ^27^Al MAS NMR spectra of samples with and without iron shows that iron helps to maintain a fraction of Al(V) species in the material. This is especially evident in the samples with the largest fraction of iron, D-0.05Fe-500 and D-0.05Fe-500-Cu. The addition of copper, on the other hand, influences the ratio between Al(IV) and Al(VI) in favor of Al(IV) for all samples calcined at 500 °C, with the exception of the D-0.005Fe-500-Cu sample (Figure 5).

#### 3.2.3 X-ray Absorption Spectroscopy (XAS)

##### 3.2.3.1 Fe and Cu K-Edge X-ray Absorption Near Edge Structure (XANES)

Normalized Fe and Cu K-edge XANES spectra, together with the spectra of the corresponding Fe and Cu reference compounds, are presented in Figure S9. Different local environments of the cation result in different K-edge profiles and pre-edge lines in the XANES spectra. The energy position of the absorption edge and the pre-edge features are correlated with the valence state of the absorbing atom in the sample. With an increasing oxidation state, each absorption feature in the XANES spectrum is shifted to higher energies.^26,38−40^

The Fe and Cu K-edge XANES analysis is used to determine the valence state and local symmetry of Fe and Cu cations in Cu and Fe functionalized alumina catalysts. The shape and energy position of the Cu K-edge in all catalysts is practically identical and coincides with those of Cu^2+^ reference compounds in which Cu^2+^ cations are located at the center of the Jahn–Teller distorted octahedron of six O atoms (Figure S9a),^38,40^ which clearly indicates that all Cu cations in the catalysts are in the divalent form octahedrally coordinated with six oxygen atoms.

The Fe K-edge profiles of the catalyst samples (Figure S9b) are very similar but not identical to one another. They all exhibit a weak pre-edge peak at 7114 eV, characteristic of tetrahedrally coordinated Fe cations lacking an inversion center, as in the case of the reference FePO_4_ sample. The energy position of the Fe K-edge in all catalysts is identical, coinciding with the edge position of the reference Fe_2_O_3_ compound. The XANES results, therefore, show that all Fe cations in the catalyst samples are in the trivalent form, partly with tetrahedral and partly with octahedral coordination to oxygen atoms in the nearest coordination shell. A linear combination fit of XANES spectra of the samples with XANES profiles of FePO_4_ as the reference for tetrahedrally coordinated Fe^3+^, and Fe_2_O_3_ as the reference for octahedrally coordinated Fe^3+^ cations, indicates that catalyst samples with Cu contain a relatively higher amount of tetrahedrally coordinated Fe^3+^ cations than the corresponding samples without Cu, calcined at the same temperature.

##### 3.2.3.2 Fe and Cu K-Edge Extended X-ray Absorption Fine Structure (EXAFS)

**Figure 6:**
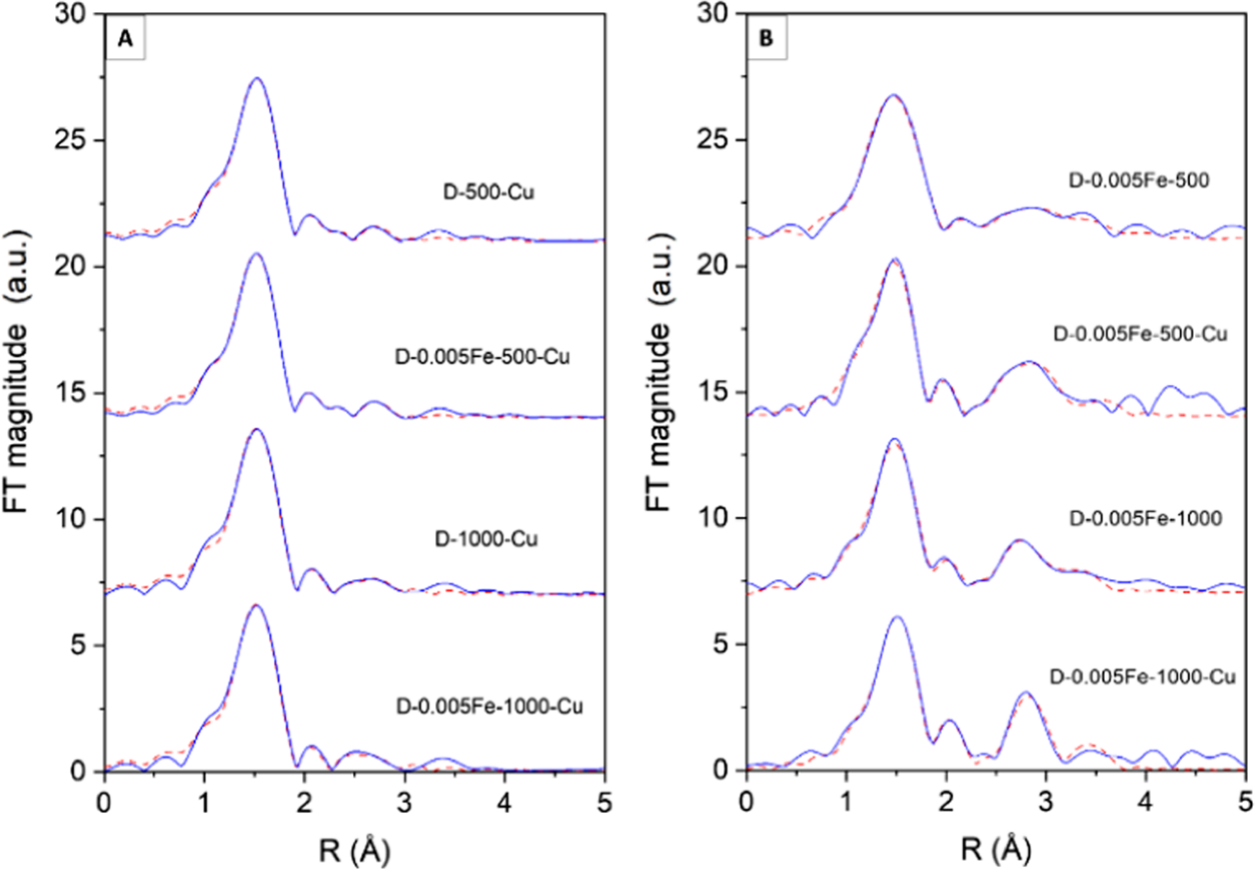
(A) Fourier transform magnitude of k^3^-weighted Cu K-edge EXAFS spectra of the Fe and Cu functionalized alumina samples, calculated in the k range of 3–13 Å^–1^. Experiment—(blue solid line); best-fit EXAFS model calculated in the R range of 1.0 to 3.1 Å—(red dashed line). (B) Fourier transform magnitude of k^3^-weighted Fe K-edge EXAFS spectra of the Fe and Cu functionalized alumina samples, calculated in the k range of 3–12 Å^–1^. Experiment—(blue solid line); best-fit EXAFS model calculated in the R range of 1.0 to 3.4 Å—(red dashed line).

Fe and Cu K-edge EXAFS analysis is used to directly probe the local structure around Fe and Cu cations in the catalysts. Fourier transforms (FT) of the k^3^-weighted Cu, and Fe K-edge EXAFS spectra of the samples are shown in Figure 6. The Cu EXAFS spectra reveal the contributions of the consecutive shells of Cu and Fe neighbors of up to approximately 4 Å. Qualitative comparisons of the Cu FT EXAFS spectra show that the average Cu neighborhood in the catalyst samples is very similar, exhibiting a strong peak at about 2 Å, which can be ascribed to photoelectron backscattering on oxygen atoms in the first Cu coordination shell, and a weak signal of more distant coordination shells. Also, the Fe FT EXAFS spectra of all samples are similar but not the same. All spectra exhibit a strong peak of the nearest oxygen coordination shell, while main structural differences are revealed in more distant coordination shells (Figure 6).

The quantitative analysis of Cu and Fe K-edge EXAFS spectra was performed with the IFEFFIT program package.^41^ Structural parameters of the average local Cu or Fe neighborhood (type and average number of neighbors, the radii, and Debye–Waller factor of neighbor shells) are quantitatively resolved from the Cu or Fe EXAFS spectra by comparing the measured EXAFS signal with the model signal, constructed *ab initio* with the FEFF6 program code,^27^ in which the photoelectron scattering paths are calculated *ab initio* from a tentative spatial distribution of Cu or Fe neighbor atoms. The atomic species of neighbors are identified in the fit by their specific scattering factor and phase shift.

In the case of Cu EXAFS spectra, excellent EXAFS fits are obtained in the k range of 3–12 Å^–1^ and in the R range of 1.0 to 3.1 Å (Figure 6a). (Structural parameters are presented in Table S1.) The results show that in all samples, Cu is coordinated to six oxygen atoms in the Jahn–Teller distorted octahedron, with four O neighbors at a distance of 1.95 Å and two O neighbors at a distance of 2.35 Å, in agreement with Cu XANES results. In the second coordination shell, Al neighbors are detected at two distances, 2.85 and 3.15 Å. In the case of samples calcined at 500 °C, we found four Al neighbors (two at shorter and two at larger distances), while in samples calcined at 1000 °C only two Al neighbors are detected (one at shorter and one at longer distances). The results indicate that all Cu atoms are directly connected to the surface of Al_2_O_3_ nanocrystals, forming Cu–O–Al bridges. After calcination at 1000 °C, about half of Cu–O–Al bridges are lost. In the process of EXAFS modeling, we checked also for the presence of CuO nanoparticles in the sample, which would be indicated by Cu neighbors at a distance of about 3.5 Å, characteristic of crystalline copper oxides or CuO nanoparticles. The presence of a smaller number of Cu neighbors at that distance (as an indication of the presence of CuO nanoparticles) cannot be completely excluded, but the EXAFS signal in the R range between 3.3 and 3.6 Å (Figure 6a) is too weak for reliable assignation. EXAFS results show that Cu cations are highly dispersed on the surface of Al_2_O_3_ nanoparticles in all samples; however, the relative number of direct Cu–O–Al bridges is significantly lower in samples calcined at 1000 °C.

In the case of Fe EXAFS spectra, a very good agreement between the model and the experimental spectra is found using the k range of 3–12 Å^–1^ and the R range of 1.0–3.3 Å (Figure 6b). (The list of best-fit parameters is given in Table S2.) In all samples, six oxygen atoms are identified in the first coordination shell, distributed at two distances: about five oxygen neighbors at 1.94 Å and about one at 2.50 Å. In more distant coordination shells, we found Fe and Al neighbors in all samples. Fe neighbors are detected at two distances characteristic of (nano)crystalline iron oxide species:^39^ on average, about one at 3.05 Å and one at 3.60 Å, while Al neighbors are identified at about 3.3 Å. The average number of Al neighbors is significantly higher in the case of the catalysts with Cu. Fe EXAFS results suggest that in all samples, iron is present in two forms. Part of Fe cations are in the form of nanostructured iron oxide clusters, and part of Fe cations are directly connected to the Al_2_O_3_ nanoparticle surface, forming Fe–O–Al bridges. The relative number of Fe–O–Al connections is higher for samples modified with Cu. A similar correlation is found in Fe XANES analysis, which indicated a relatively higher amount of tetrahedrally coordinated Fe^3+^ cations in samples with Cu, which suggests that Fe cations forming Fe–O–Al bridges are located on the surface of Al_2_O_3_ at the sites with tetrahedral symmetry. No Cu neighbors were detected in the second coordination shell around Fe cations. However, the presence of Fe–O–Cu connections is not completely excluded. The eventual contribution of Cu neighbors may be below the detection limit.

#### 3.2.4 Transmission Electron Microscopy (TEM)

**Figure 7:**
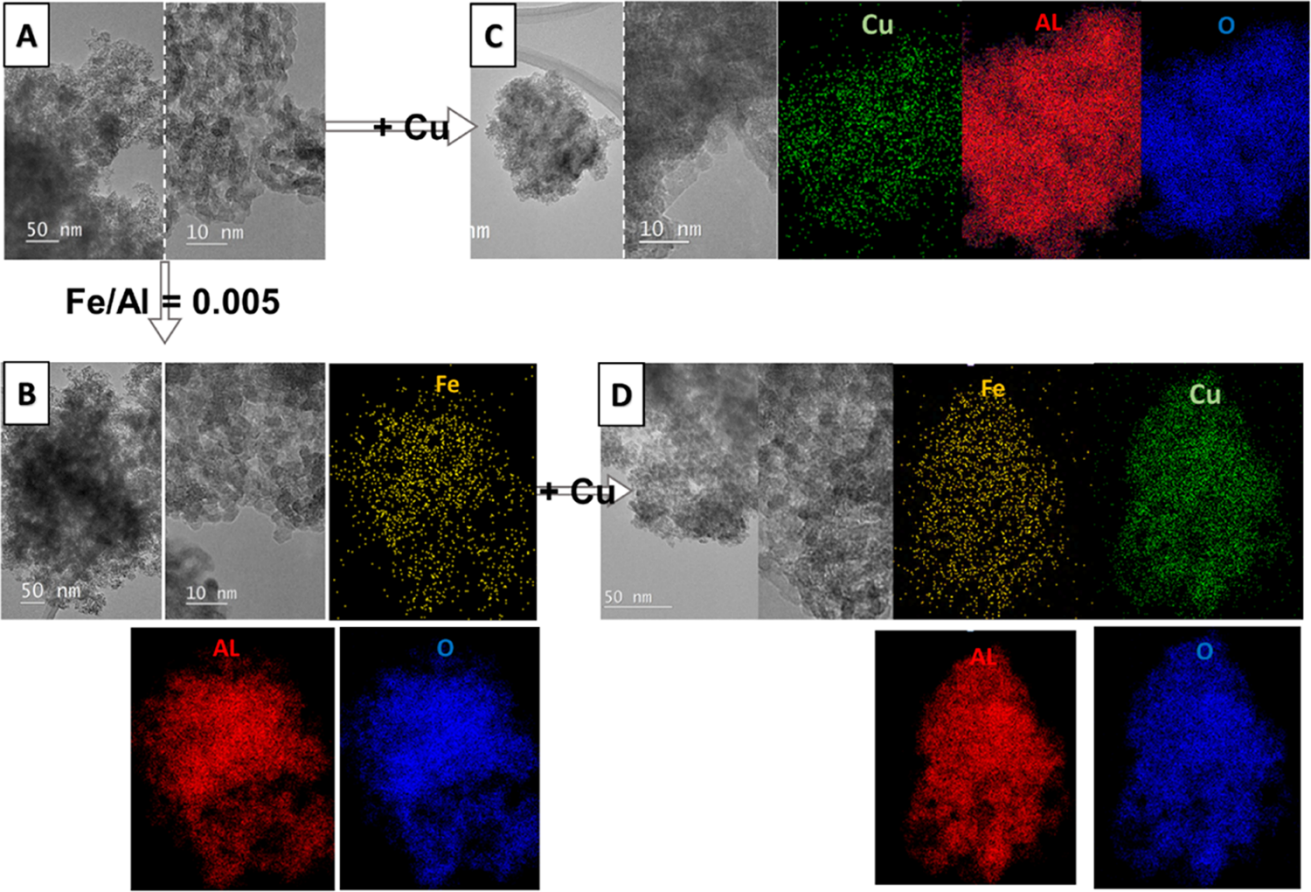
TEM images with EDAX mappings of samples calcined at 500 °C where the uniform distribution of Fe and Cu was observed in the alumina matrix. (A) D-500: TEM images of the polycrystalline alumina matrix with a particle size of 5–10 nm; (B) D-0.005Fe-500: TEM images of iron-containing alumina with EDXS mappings of Fe, Al, and O; (C) D-500-Cu: TEM images of copper-containing alumina with EDXS mappings of Cu, Al, and O; and (D) D-0.005Fe-500-Cu: TEM images of iron- and copper-containing alumina with EDXS mappings of Fe, Cu, Al, and O.

**Figure 8:**
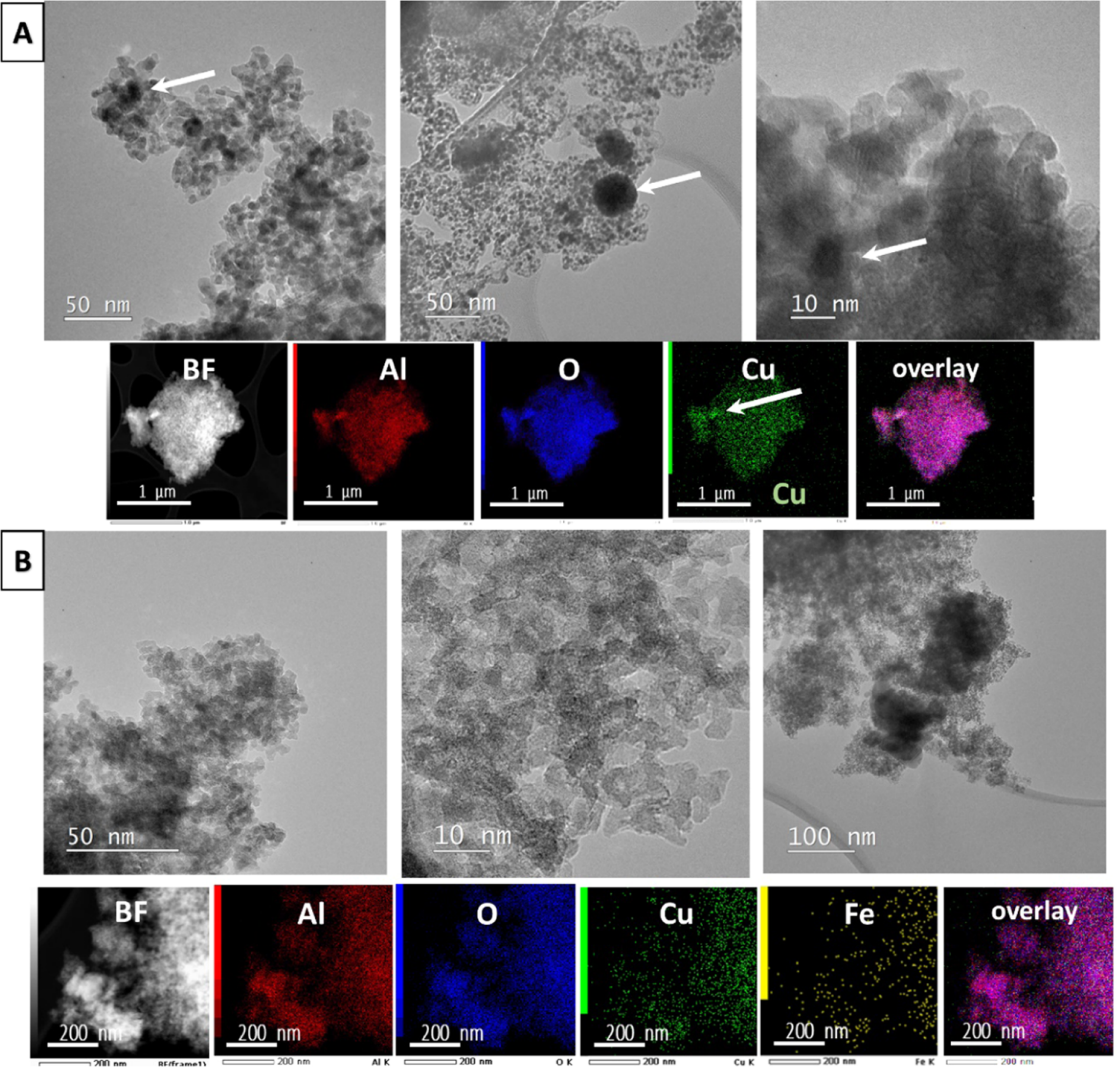
In sample D-500-Cu-R (A) after exposure to the catalytic process, small CuO nanoparticles are visible in TEM micrographs and EDXS mappings (indicated with white arrows). (B) TEM images and elemental EDXS mappings of the D-0.005Fe-500-Cu-R sample where no CuO-rich phase was detected.

TEM micrographs and elemental mappings of the most relevant catalytic materials are shown in Figures 7 and 8. Sample D-500 (Figure 7a) consists of polycrystalline pure alumina with a particle size of up to 5 nm. The small particle size observed in TEM is in accordance with low and wide diffraction maxima in XRD. Sample D-0.005Fe-500 (Figure 7b) has uniformly distributed Fe in the alumina matrix. No Fe-rich nanoparticles were observed. Sample D-500-Cu (Figure 7c) has uniformly distributed Cu in the polycrystalline alumina matrix. No Cu-rich nanoparticles were detected. Sample D-0.005Fe-500-Cu is very similar, with a uniform distribution of Cu and Fe in the matrix alumina phase with no indication of Cu and/or Fe-rich nanoparticle presence (Figure 7d).

In sample D-500-Cu-R, which has been used in a catalytic reaction, we found small nanoparticles of Cu oxide (Figure 8a, labeled with white arrows). The majority of Cu remained uniformly distributed in the alumina substrate. Sample D-0.005Fe-500-Cu-R (Figure 8b) contains uniformly distributed Fe and Cu after the catalytic process, but no Cu or Fe oxide nanoparticles were found.

**Figure 9:**
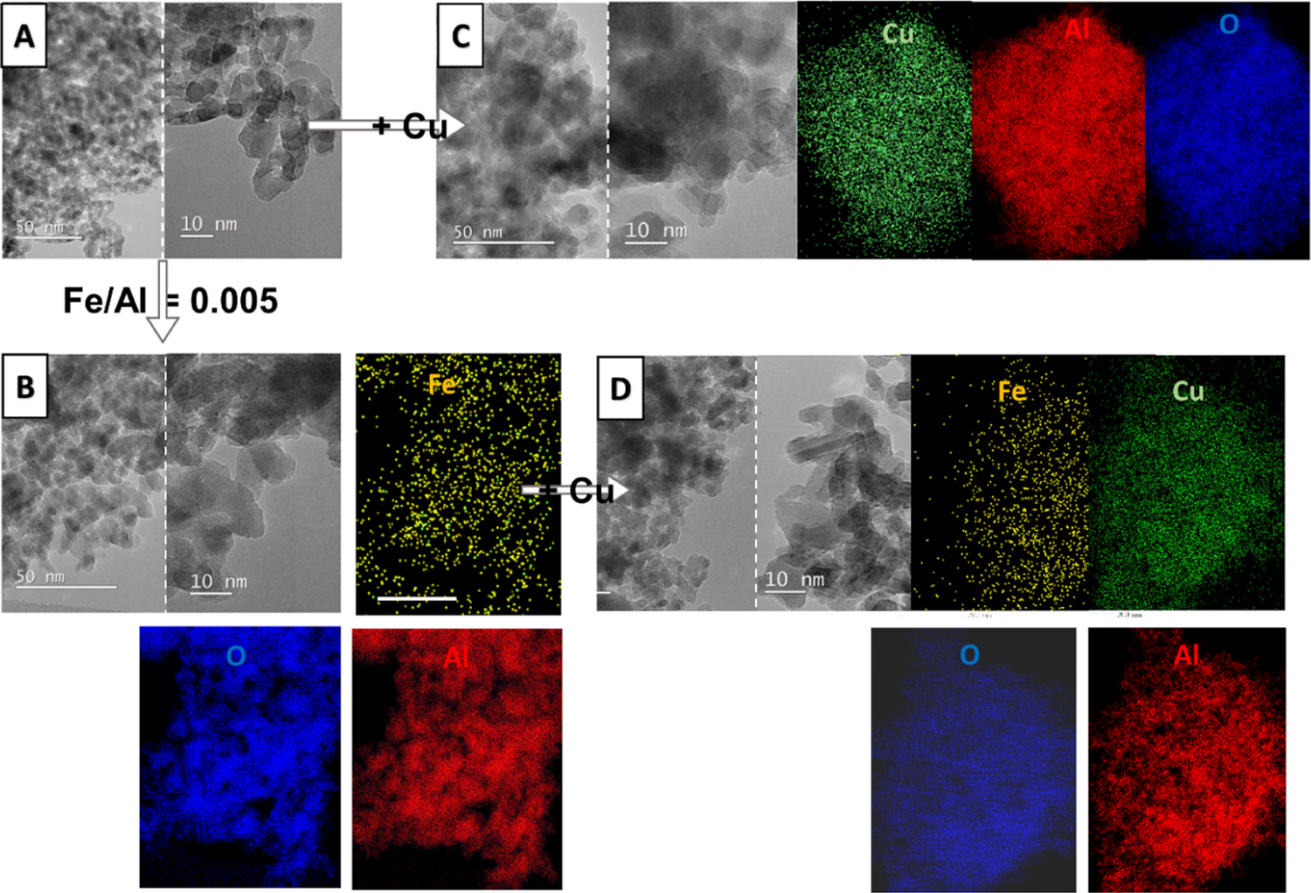
TEM images with EDAX mappings of samples calcined at 1000 °C where a uniform distribution of Fe and Cu was observed in the alumina matrix. (A) D-1000: TEM images of the polycrystalline alumina matrix with a particle size of 5–10 nm; (B) D-0.005Fe-1000: TEM images of iron-containing alumina with EDXS mappings of Fe, Al, and O; (C) D-1000-Cu: TEM images of copper-containing alumina with EDXS mappings of Cu, Al, and O; and (D) D-0.005Fe-1000-Cu: TEM images of iron- and copper-containing alumina with EDXS mappings of Fe, Cu, Al, and O.

Samples calcined at 1000 °C are displayed in Figure 9. The sample D-1000 consists of polycrystalline alumina with a particle size of 5–10 nm (Figure 9a). In Figure 9b, TEM micrographs and EDXS mappings of sample D-0.005Fe-1000 are shown, where the uniform distribution of Fe in the alumina matrix was observed. In Figure 9c, the TEM images and EDXS mapping of sample D-1000-Cu are displayed. Cu is uniformly distributed inside the polycrystalline alumina matrix. TEM and EDXS elemental mappings of sample D-0.005Fe-1000-Cu, where a uniform distribution of Cu and Fe are found in the alumina matrix, are presented in Figure 9d. No CuO nanoparticles were found with TEM in any of these samples.

### 3.3 Catalytic Performance

**Figure 10:**
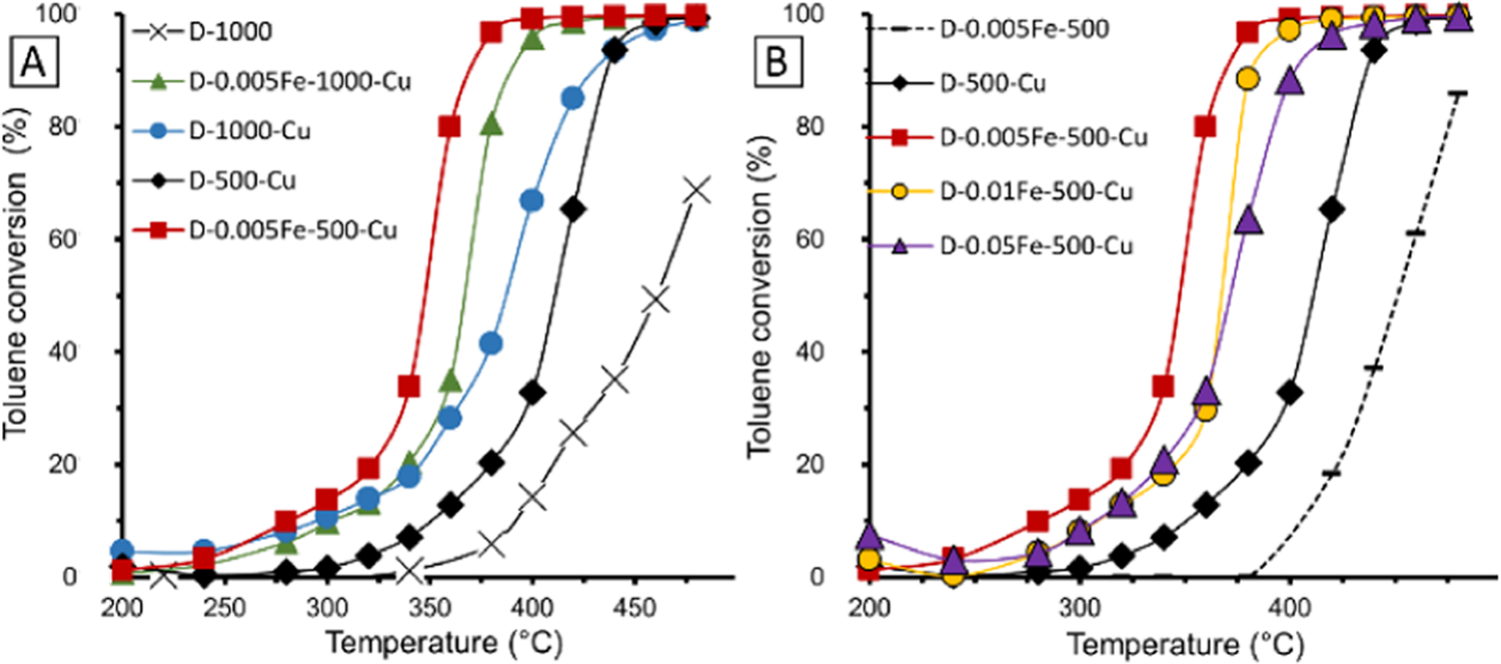
Catalytic activity measured in a temperature-programmed regime: (A) influence of calcination temperature and (B) influence of iron content (with lines for clarity).

The total toluene oxidation reaction was chosen as a model reaction to study the activity of prepared catalysts. The activity of samples with different iron contents (Figure 10b) shows that the most active sample was that with the 0.005Fe/Al molar ratio, which reached *T*_90_ (temperature at which toluene conversion equals 90%) at 372 °C. Catalysts that did not contain copper (D-0.005Fe-500) performed very poorly with *T*_50_ at 460 °C. This confirms that the presence of copper is crucial for catalytic activity. Regardless of the Fe content, the Cu-containing catalysts outperformed the catalysts prepared without iron (D-500-Cu). The observed activity order, determined by *T*_90_, based on the Fe/Al ratio, was 0 < 0.05 < 0.01 < 0.005. The difference between the samples with Fe/Al ratios of 0.01 and 0.05 started to appear above 360 °C.

Materials calcined at 1000 °C were also tested (Figure 10a). The metal-free support D-1000 displayed poor activity. With addition of copper, the activity improved, and the catalyst D-1000-Cu was even more active compared to sample D-500-Cu. The D-0.005Fe-1000-Cu catalyst performed well and proved to be more active than the sample prepared without iron. Nonetheless, the activity was slightly lower compared to the D-0.005Fe-500-Cu catalyst.

**Figure 11:**
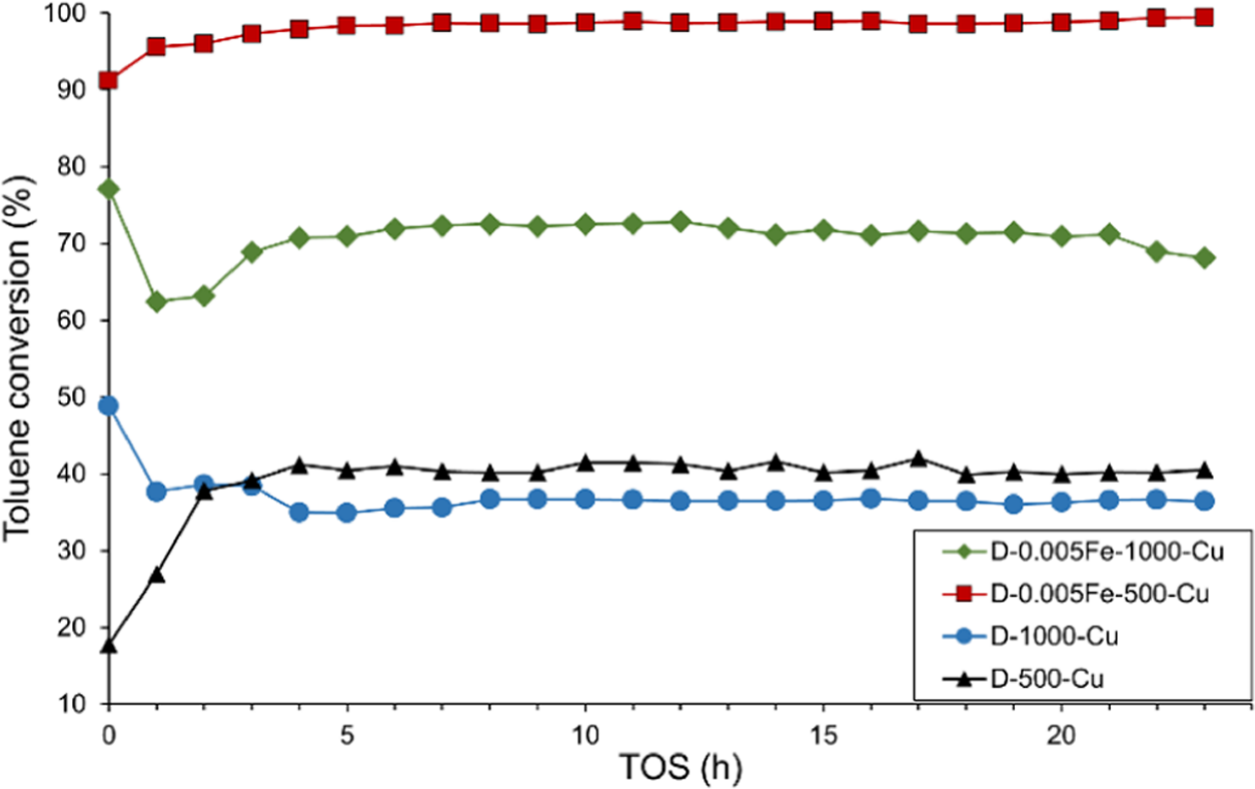
Time on stream (TOS) runs carried out at 380 °C for a 23 h period.

Stability of the catalysts D-500-Cu, D-1000-Cu, D-0.005Fe-500-Cu, and D-0.005Fe-1000-Cu were studied under isothermal conditions at 380 °C (Figure 11). After the initial induction period of about 2 h, all catalysts exhibited stable catalytic activity for the remainder of the 23 h experiment. This was confirmed with the structural integrity analyzed by the in situ XRD analysis of the D-0.005Fe-500-Cu catalyst during repetitive heating–cooling cycles, which showed no identifiable structural changes (Figure S10).

**Figure 12:**
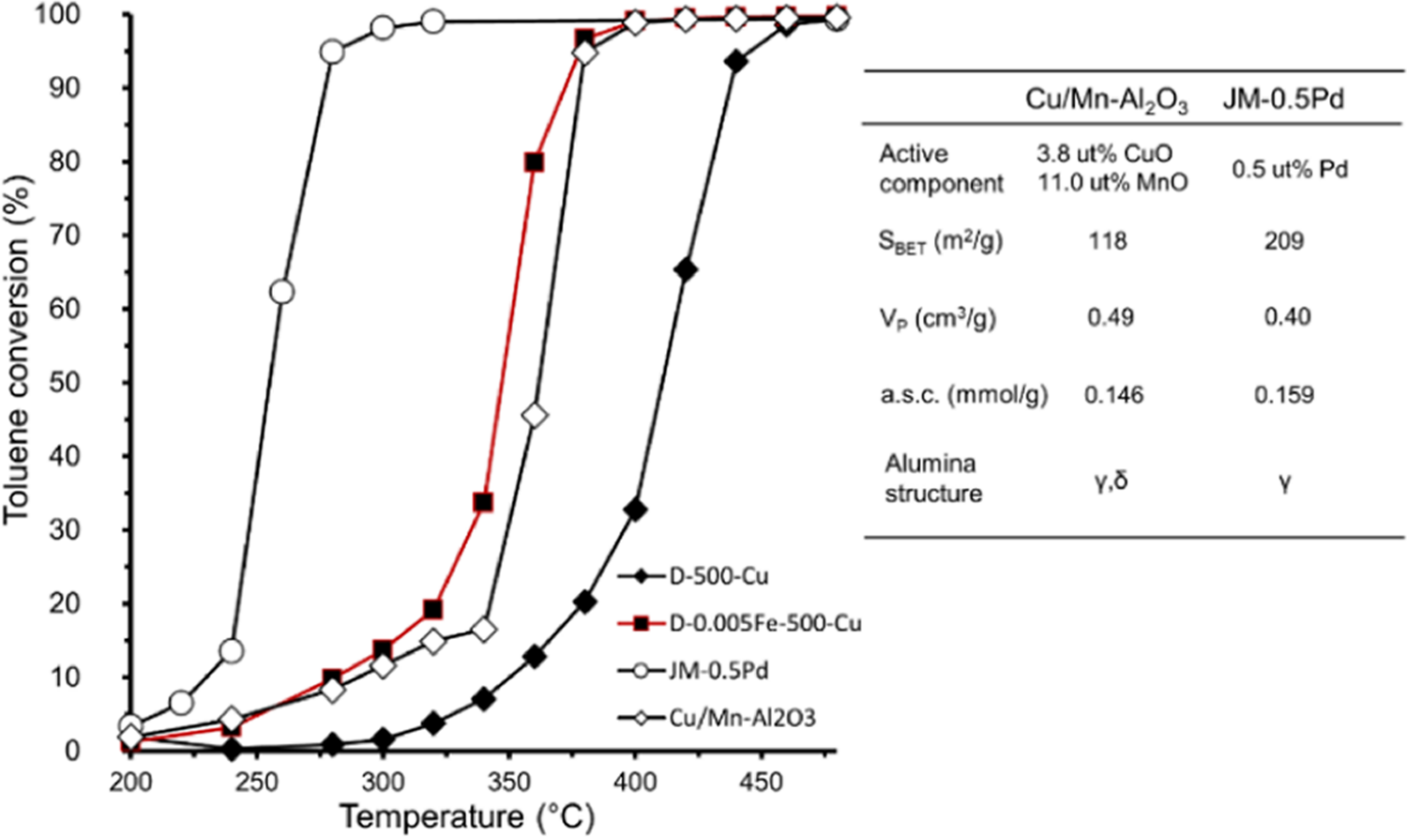
Comparison of the catalytic activity for the toluene oxidation reaction between two commercial catalysts (JM-0.5Pd and Cu/Mn–Al_2_O_3_) and samples D-500-Cu and D-0.005Fe-500-Cu. Presented is also some basic information like *S*_BET_, *V*_P_, a.s.c., active compound content, and the support alumina crystal structure of the two commercially available catalysts.

Figure 12 compares the catalytic activity of two commercially available samples, JM-0.5Pd and Cu/Mn–Al_2_O_3_, with materials developed in this research. The activity of the noble metal-containing catalyst (JM-0.5Pd) is unmatched, as it reaches almost full conversion of toluene at only 280 °C. The other sample, Cu/Mn–Al_2_O_3_, performs fairly similarly to the bimetallic D-0.005Fe-500-Cu: they both reach almost full conversion of toluene at 380 °C. Even more, the D-0.005Fe-500-Cu achieves higher conversions at temperatures between 320 and 360 °C. Sample D-500-Cu is shown for comparison of the activity increase between the iron-free sample and the former mentioned 0.005Fe/Al-containing sample.

### 3.4 Determination of Redox Properties and Reactivity of the Catalyst Oxygen Species

#### 3.4.1 Temperature-Programmed Reduction (H_2_-TPR)

**Figure 13:**
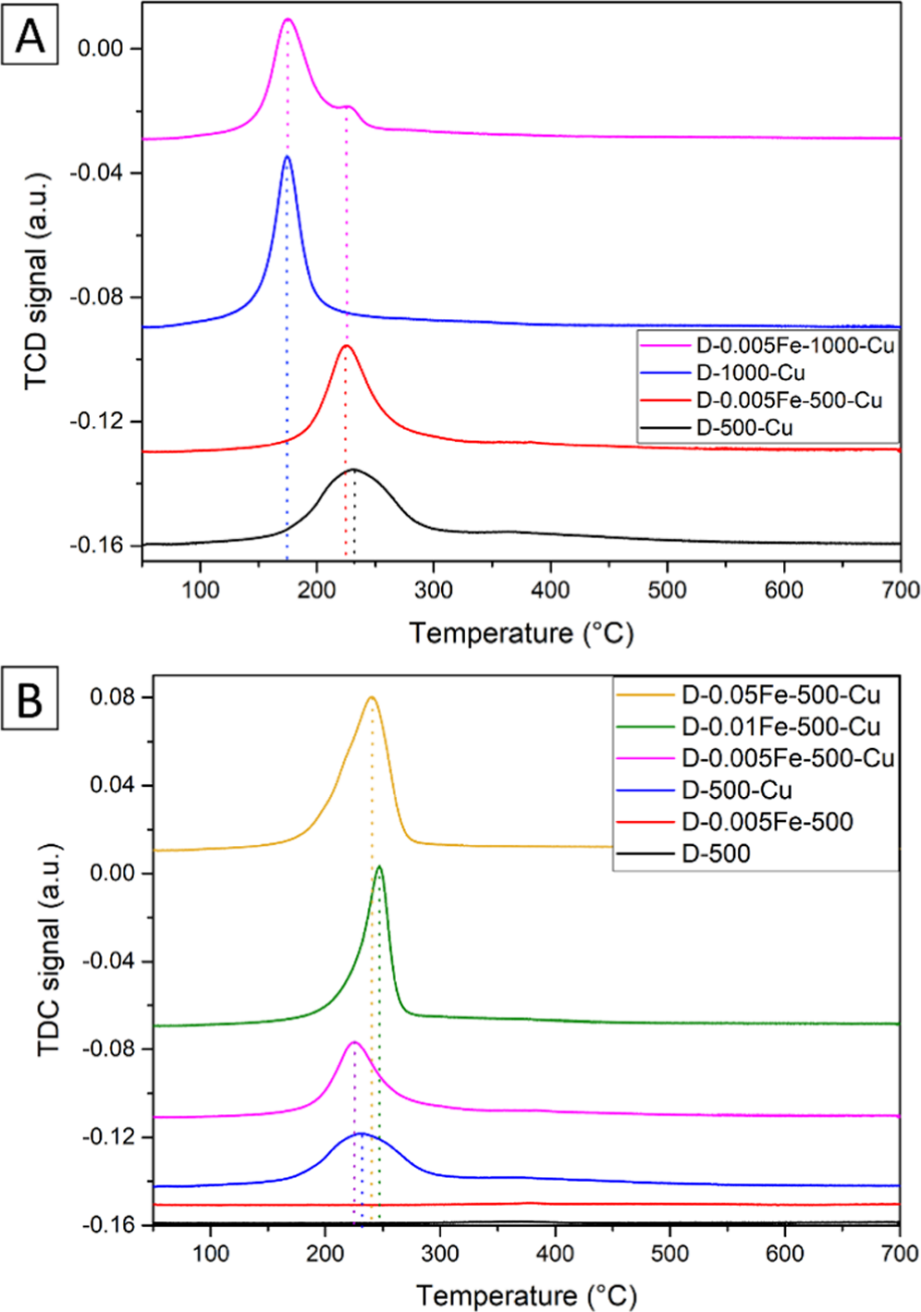
Temperature-programmed reduction (TPR) profiles with hydrogen. (A) Samples calcined at 500 and 1000 °C, prepared without Fe and with an Fe/Al ratio of 0.005. (B) Reduction profiles of samples with different iron contents and samples D-500 and D-0.005Fe-500, prepared without Cu. The profiles are offset vertically for clarity.

Figure 13a shows reduction profiles of Cu-containing catalysts prepared from materials with an Fe/Al molar ratio of 0.005 and without Fe, calcined at 500 and 1000 °C, respectively. Sample D-0.005Fe-1000-Cu has a bimodal reduction distribution with the apex of the first reduction peak at about 170 °C. The peak asymmetry of the sample D-0.005Fe-500-Cu is displayed with a tail on the high-temperature side. The reduction peak maximum occurs at 225 °C in the case of this sample. The sample D-500-Cu apparently possesses a broader size distribution of copper particles that is visible as a wider reduction profile of this sample in comparison to the sample with the 0.005Fe content. This second peak has its maximum at the same temperature (225 °C) as the sample D-0.005Fe-500-Cu indicating a correlation between the two samples in terms of redox-active components. To summarize, calcination of the alumina support at 1000 °C produces more easily reducible copper species, which is also in line with the decreased number of Cu–O–Al chemical bonds estimated from EXAFS analysis.

The reduction profiles were also recorded on samples D-500 and D-0.005Fe-500, in which no copper was present (Figure 13b). Their reduction profiles were almost flat, which implies that the redox activity of iron-containing phases is negligible. This figure also presents reduction profiles of catalyst samples with a progressively increasing Fe/Al ratio (0.005–0.05). Interestingly, the catalytically most active material (D-0.005Fe-500-Cu) has the lowest reduction peak maximum at 225 °C and is the only one with a shoulder on the high-temperature side. The samples with a higher Fe content have their reduction peak maximum at 240 (D-0.05Fe-500-Cu) and 248 °C (D-0.01Fe-500-Cu), which is shifted to higher temperatures by about 10–20 °C compared to samples with the lowest amount of iron.

The total hydrogen consumption order for the samples presented in Figure 13b was the following: D-0.05Fe-500-Cu < D-500-Cu < D-0.005Fe-500-Cu < D-0.01Fe-500-Cu < D-0.005Fe-500 < D-500. The ratio between the actual and theoretical hydrogen consumption for individual samples (Table 2) shows a clear decreasing trend: an increasing presence of iron results in lower reducibility of copper species. Apparently, there exists some Fe-induced interaction with copper clusters, making them less susceptible to reduction, in the case of the alumina calcination at 1000 °C. There is also less removable oxygen available at the Fe/Al molar ratio of 0.005 when calcined at 500 °C in comparison to 1000 °C (68 and 58%, respectively).

**Table 2:** Temperature at 50% of the Conversion of Toluene (*T*_50_) and Temperature at 90% of Toluene Conversion (*T*_90_)^a^

sample	*T*_90_ (°C)	*T*_50_ (°C)	H_2_-TPR (μmol g^–1^)	H_2_ consumption/theoretical H_2_ consumption (%)
D-500			21	
D-500-Cu	437	411	947	78
D-1000-Cu	432	387	883	79
D-0.005Fe-500		460	60	44
D-0.005Fe-500-Cu	372	347	850	68
D-0.005Fe-1000-Cu	392	366	760	58
D-0.01Fe-500-Cu	380	367	692	47
D-0.05Fe-500-Cu	403	372	972	39

a Hydrogen consumption during the H_2_-TPR analysis and fraction of the actual/theoretical hydrogen consumption.

#### 3.4.2 Temperature-Programmed Desorption of Oxygen (O_2_-TPD)

**Figure 14:**
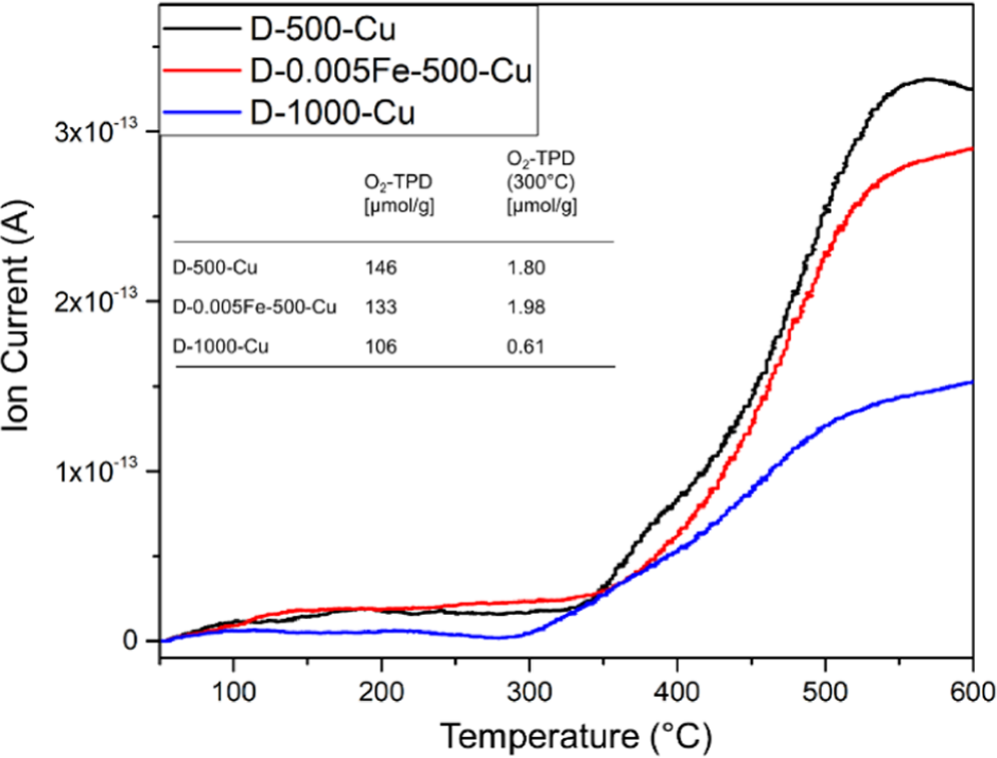
TPD-O_2_ profiles obtained during the examination of selected catalyst samples with an inset table of the total oxygen evolution (O_2_-TPD) and oxygen evolution in TPD up to 300 °C.

Temperature-programmed desorption of oxygen was recorded to investigate the role of adsorbed and lattice oxygen for the catalytic activity of prepared catalysts. As can be seen in Figure 14, almost all desorbed oxygen can be ascribed to be of lattice origin based on the high temperature required for its removal (above 300 °C). A lower amount of available oxygen (by almost 30%) is observed in the sample D-1000-Cu and by 9% in D-0.005Fe-Cu-500 in comparison to D-500-Cu. The desorption of oxygen starts the earliest in sample D-1000-Cu at 300 °C, whereas, in the case of the other two analyzed samples, it begins at around 350 °C. The obtained desorption profiles also indicate the presence of a shoulder at 380 °C in iron-free samples, which could indicate a small amount of oxygen desorbed from the surface.

An interesting observation was made when only the oxygen desorption at low temperatures (below 300 °C) was examined, which is attributed to the adsorbed surface oxygen species. In this case, the sample D-0.005Fe-500-Cu desorbed the highest amount of oxygen, whereas the sample D-1000-Cu desorbed by far the lowest oxygen amount, more than 3 times less. The sample D-500-Cu desorbed approximately 10% less O_2_ at 300 °C in comparison to D-0.005Fe-Cu-500.

#### 3.4.3 Temperature-Programmed Desorption/Reaction of Toluene (TPD/R of Toluene)

**Figure 15:**
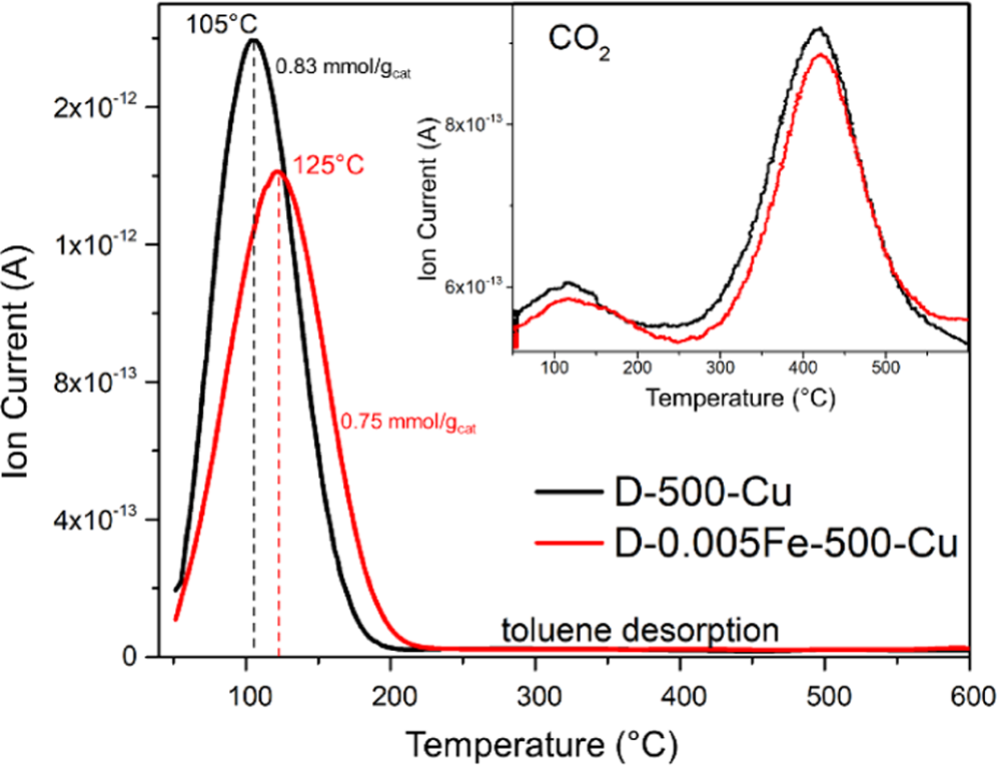
TPD profiles of toluene obtained during the TPD/R-toluene analysis for various catalysts. Inset is the corresponding TPD spectra of CO_2_ obtained in the temperature-programmed regime after saturation of the catalyst surface with toluene.

The temperature-programmed desorption/reaction of toluene was analyzed to determine the toluene adsorption/desorption affinity and the role of lattice oxygen for the toluene oxidation reaction. Almost all of the adsorbed toluene was released from the samples D-500-Cu and D-0.005Fe-500-Cu below 220 °C (Figure 15). The amount of toluene that was desorbed in the case of sample D-0.005Fe-500-Cu was also 10% lower in comparison to the sample prepared without iron. An interesting observation was made by measuring the CO_2_ temporal profile (Figure 15-inset); both catalysts displayed significant CO_2_ formation. The first peak was observed in the temperature region in which the majority of toluene was desorbed. This indicates that a fraction of toluene reacted with oxygen that is easily available, causing its total oxidation to CO_2_. The second, a more pronounced peak of CO_2_ started above 300 °C and reached its maximum at around 420 °C in both samples. This indicates the presence of two types of oxygen species (more reactive and less abundant and more abundant but less reactive), which are responsible for toluene oxidation to CO_2_ and water in two distinct temperature windows, 50–250 and 300–450 °C, respectively. Except for CO_2_ and H_2_O (trace not shown), no other reaction products of toluene oxidation were observed.

#### 3.4.4 Pulsed Toluene Oxidation Reaction

The analysis was performed on the D-500-Cu sample, as it exhibited the highest redox performance established through H_2_-TPR, O_2_-TPD, and toluene TPR/D. As a result, it is reasonable to expect the highest contribution to toluene oxidation via lattice oxygen from this sample. It can be seen in Figure S11 that when the reaction was carried out in an inert atmosphere, a negligibly small amount of CO_2_ was formed. On the contrary, when the pulsed toluene oxidation reaction was conducted in an air atmosphere, a noticeable (i.e., by more than 2 orders of magnitude higher) formation of CO_2_ was observed (Figure S11b). This clearly shows that in the given range of operating and reaction conditions, lattice oxygen only marginally contributes to the conversion of toluene and that the reaction proceeds via continuously adsorbing molecular oxygen (Langmuir–Hinshelwood mechanism).

Among the reaction products monitored during the described experiments, only the formation of CO_2_ and H_2_O was observed. Furthermore, adsorption of toluene (and partially oxidized reaction species) on the catalyst surface was observed when the pulsed toluene oxidation reaction was performed in an inert atmosphere. This was confirmed by CHNS elemental analysis, which showed that the carbon content on the catalyst surface increased by 0.3 wt % after 20 pulses of toluene. On the other hand, a negligible small accumulation of the carbon-containing species on the catalyst surface was observed when the reaction was carried out in an air atmosphere.

## 4 Discussion

The prepared catalysts are complex multicomponent materials with several properties (i.e., affinity for toluene adsorption, redox chemistry of metal oxide clusters, and ability for molecular oxygen activation), which in a concerted manner contribute to toluene oxidation activity.

The presence of Lewis acid sites and sufficiently small pore sizes are reported as important factors influencing toluene adsorption through acid–base interactions and secondary interactions such as van der Waals forces between the support and toluene.^42^

Iron is present as Fe^3+^ with tetrahedral and octahedral coordination to oxygen as nanostructured oligomeric Fe-oxo clusters, and part of Fe cations are directly connected to the Al_2_O_3_ nanoparticle surface, forming Fe–O–Al bridges. A relative number of Fe–O–Al connections is higher for samples modified with Cu. Copper is present as Cu^2+^ in the Jahn–Teller distorted octahedron, directly connected to the alumina surface via Cu–O–Al bridges. Such undercoordinated surface sites are recognized as adsorption sites in catalytic reactions due to a lower “degree of bond saturation” in comparison to ideal crystals.^43^ By increasing the iron content and thus decoration of the alumina support, clustering and growth of copper oxide clusters is promoted, as can be observed from UV–vis analysis.

In the most active D-0.005Fe-500-Cu catalyst, the CuO_6_ octahedra are at the interface with the alumina carrier bound to 4 Al atoms, whereas in the less active D-0.005Fe-1000-Cu, copper-oxo octahedra are bound only to 3 Al atoms. The Cu-containing catalysts prepared on a more ordered support (δ/θ phase, calcined at 1000 °C, Figure S3) are reduced somewhat easier in comparison to the ones based on γ-Al_2_O_3_. This occurs due to a fewer number of Cu–O–Al bonds, as identified via XAS analysis. Also, with increasing Fe content, the fraction of reducible copper species progressively decreases.

Interestingly, only a small amount of iron (0.005Fe/Al molar ratio) performed best in this studied reaction. Copper is clearly the main contributor to catalytic activity, as all of the catalysts without Cu are significantly less active. No direct Cu–O–Fe bridges were detected by Cu or Fe EXAFS analysis, but their presence cannot be completely excluded since their signal may be below the detection limit of the EXAFS analysis.

The Fe–Cu synergy was observed indirectly through altered redox chemistry probed by H_2_-TPR and structural UV–vis analysis. As also evidenced by UV–vis spectroscopy, a reduction in the energy gap of Al_2_O_3_ occurs (Figure S13). The results show that a part of iron is embedded in the Al_2_O_3_ crystal lattice at Al sites and thus creates additional electronic states in the Al_2_O_3_ crystal structure within the energy gap below the conduction band. These electronic states extend over the entire crystal surface and act on Cu, which is bound to the Al_2_O_3_ surface. Cu binds to the surface after Fe is already incorporated into Al_2_O_3_.

Higher activity for the toluene oxidation reaction was observed in all iron-containing samples in comparison to the catalysts without Fe. We were unable to establish a correlation between the catalytic and redox activity of tested materials via H_2_-TPR, O_2_-TPD, and TPD/R of toluene. These analyses involve probing mainly lattice oxygen and also adsorbed O species. However, about 2 orders of magnitude more CO_2_ was formed upon toluene oxidation in oxygen compared to the inert atmosphere, clearly identifying a minor role of lattice oxygen in catalytic oxidation. As a result, the toluene oxidation mechanism takes place mainly via molecular/atomic oxygen continuously formed on the surface during a reaction. This parallel reaction pathway is supported by the fact that the redox-inactive Fe–Al_2_O_3_ sample (Figure 10b) exhibits some catalytic activity, albeit much lower than bimetallic (CuFe) ones.

The optimal toluene oxidation activity, which was observed on a lightly promoted D-0.005Fe-500-Cu catalyst, likely originates from the most facile dioxygen activation over finely dispersed copper oxide clusters anchored to the electronically modified alumina support via Cu–O–Al bonding.

## 5 Conclusions

Dawsonite originating γ or θ/δ alumina supports were synthesized as a consequence of calcination at 500 (γ-alumina) or 1000 °C (θ-alumina and δ-alumina) and modified with iron in an Fe/Al molar ratio from 0 to 0.05. Addition of copper (8 wt % of CuO) resulted in the copper present in Jahn–Teller distorted octahedra and connected to the surface with Cu–O–Al bridges. The alumina phase influenced the relative number of copper-oxo bridges and the type of binding to the support, which has an effect on reduction taking place at higher temperatures. A higher fraction of iron in the alumina support progressively decreases the redox activity of copper, which is manifested as a decreasing amount of metallic copper achieved during reduction. When tested for catalytic activity in the toluene oxidation reaction, the nature of the metal–support bonding and the optimal abundance between Cu–O–Al and Fe–O–Al species in the catalysts seem to be the origin of the observed catalytic activity of the D-0.005Fe-500-Cu (dawsonite originating γ alumina support synthesized as a consequence of calcination at 500 °C) at a lower temperature. Namely, the toluene oxidation activity is promoted but very strongly dependent on the molar fraction of iron, which should be maintained around 0.005. The total toluene oxidation mainly occurs via participation of molecular oxygen adsorbed on the catalyst, with minor participation of the lattice oxygen and redox chemistry of the CuO*_x_* species.

Understanding the structure–property–activity relationship of the designed bimetallic oxide catalysts on the γ-alumina support is extremely important not only for the described targeted application for air pollution prevention but also for other applications in heterogeneous catalysis, green chemistry, advanced manufacturing, and furthermore in electrocatalysis and photocatalysis.

## Supporting Information Available

Reactor scheme; synthesis; XRD of samples calcined at 1000 °C; SEM images; XRF elemental analysis; pyridine TPD; N_2_ physisorption; NMR; XAS (XANES and EXAFS); XRD analysis of repeated heating and cooling of catalysts; pulsed toluene oxidation reaction; CHN elemental analysis; and band gap analysis (PDF)
