# Supplementary material for: Winning Combination of Cu and Fe Oxide Clusters with an Alumina Support for Low-Temperature Catalytic Oxidation of Volatile Organic Compounds
Source: ACS Appl Mater Interfaces. 2023 Jun 2;15(23):28747–62. doi: 10.1021/acsami.3c02705 (PMC10273233; doi:10.1021/acsami.3c02705)
Supplement: Supplementary file 1 — am3c02705_si_001.pdf [file am3c02705_si_001.pdf]

Supporting Information

A winning combination of Cu and Fe oxide clusters with alumina support for low temperature catalytic oxidation of volatile organic compounds

*Tadej Žumbar[a], Iztok Arčon[b,c], Petar Djinović[a,b], Giuliana Aquilanti[d], Gregor Žerjav[a], Albin Pintar[a], Alenka Ristić[a], Goran Dražić[a], Janez Volavšek[a], Gregor Mali[a], Margarita Popova[e], Nataša Zabukovec Logar[a,b], Nataša Novak Tušar[a,b,] **

[a]National Institute of Chemistry, Hajdrihova 19, SI-1001 Ljubljana, Slovenia

[b]University of Nova Gorica, Vipavska 13, 5000 Nova Gorica, Slovenia

[c]Jožef Stefan Institute, Jamova 39, SI-1000 Ljubljana, Slovenia

[d]Elettra-Sincrotrone Trieste S.C.p.A. Strada Statale 14 - km 163,5 in AREA Science Park, 34149 Basovizza, Trieste Italy

[e]Institute of Organic Chemistry with Centre of Phytochemistry, Bulgarian Academy of Sciences, Acad. G. Bonchev Str., Bl. 9, 1113 Sofia, Bulgaria

Corresponding author email: natasa.novak.tusar@ki.si

Reactor scheme

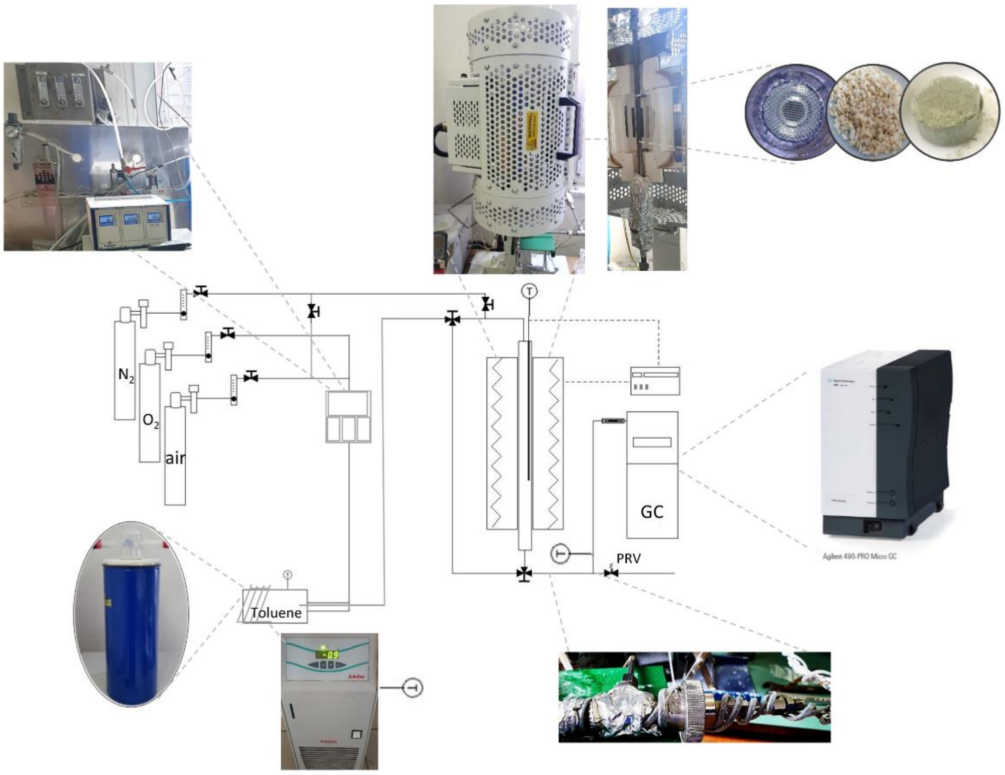

Figure S1. Reactor scheme used for determination of catalytic activity and stability.

Synthesis

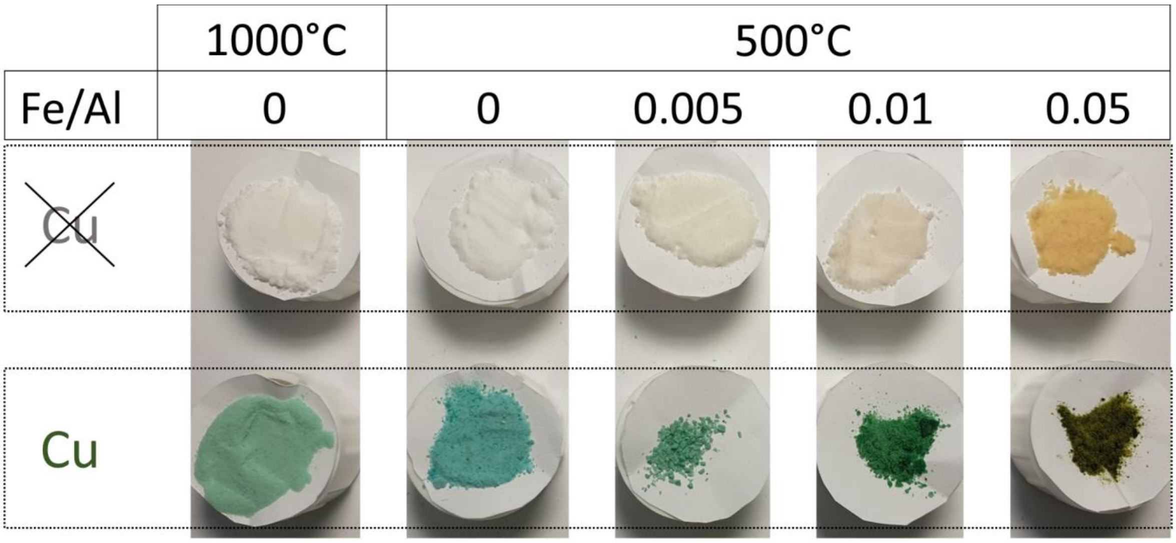

Figure S2. Samples with different Fe/Al molar ratio, with and without copper, and calcined at 500 and 1000°C.

XRD of samples calcined at 1000 °C

X-ray diffraction analysis of the samples showed a mixture of δ (PDF 00-046-1131) and θ (PDF 01-086-1410) Al_2O_3 phase. No copper or iron oxides were detected with XRD.

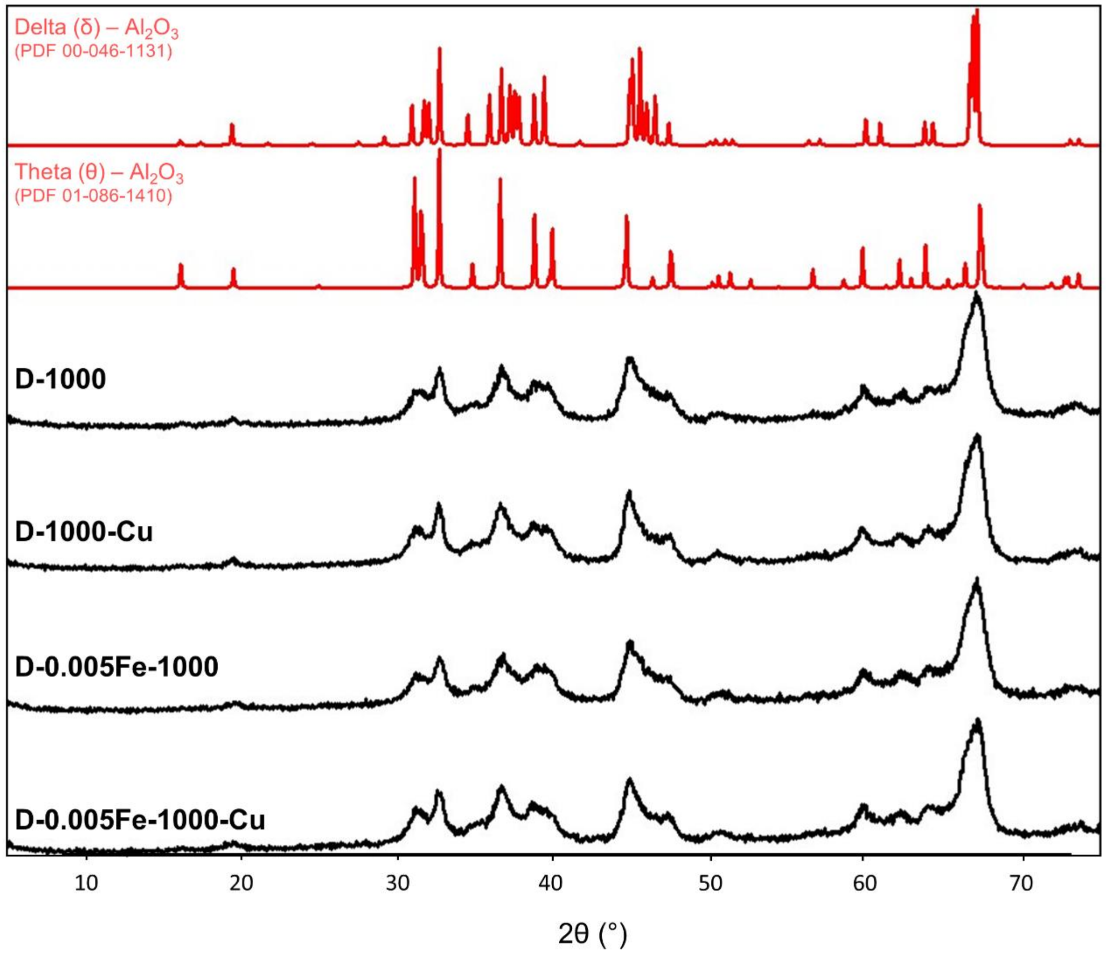

Figure S3. XRD patterns of samples prepared at 1000 °C, with and without Cu. Reference patterns of delta and theta alumina are presented in red.

SEM

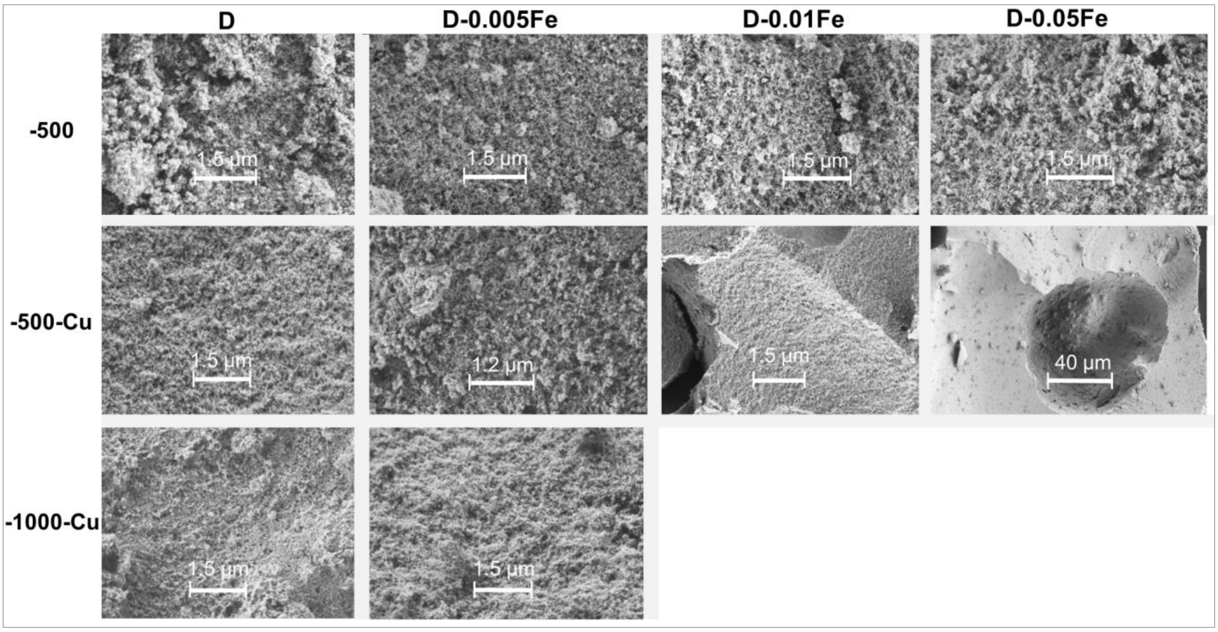

Figure S4. SEM images of samples calcined at 500°C and copper containing samples prepared at 500 and 1000°C, with different iron content.

XRF analysis

Samples were dried and calcined prior to the analysis to determine the total weight loss of the material during the following sample preparation steps with Phoenix R sample preparation unit and lithium tetraborate flux (type LT100 Granular) addition in ratio 1:8 (sample: flux). Results were analyzed with Spectra plus Measurement and Launcher V 2.2.47 software and two different calculation methods were implemented to avoid any miscalculations. The loss of ignition was identified as water loss and accounted in the total mass of the sample for correct data analysis.

Determination of surface acidic properties (pyridine-TPD)

Figure S5a presents the difference in strength of acid sites with increased calcination temperature (500 and 1000°C) in one iron free and one iron containing sample. Catalyst with Fe/Al molar ratio of 0.005 was chosen as it was the most active during the catalytic tests *(vide*

infra). In both cases, the first desorption peak of pyridine is observed at around 240°C and this peak is larger in the samples calcined at 500°C, revealing presence of a larger number of relatively weak acid sites. Low concentration of iron does not influence the strength of acid sites in most cases. Catalysts prepared at 1000°C contained stronger acid sites, based on more pronounced pyridine desorption at temperatures above 350°C, although their total concentration was reduced by about 50% in respect to those prepared at 500°C, which is likely related to specific surface area loss, which is also about 50% (Table 1).

The pyridine desorption profiles are compared for different Fe loadings on alumina calcined at 500°C with and without copper (Figure S5b). Presence of only copper decreases the total acid site abundance, which is consistent with lower intrinsic acidity of CuO compared to Al_2O_3 (REFERENCE https://doi.org/10.1002/anie.200803837). However, when iron amount is gradually increased in the bimetallic Cu-Fe materials containing a constant Cu loading, we could observe a progressive trend in increasing weak (pyridine desorption peak at about 240°C) and strong acid.

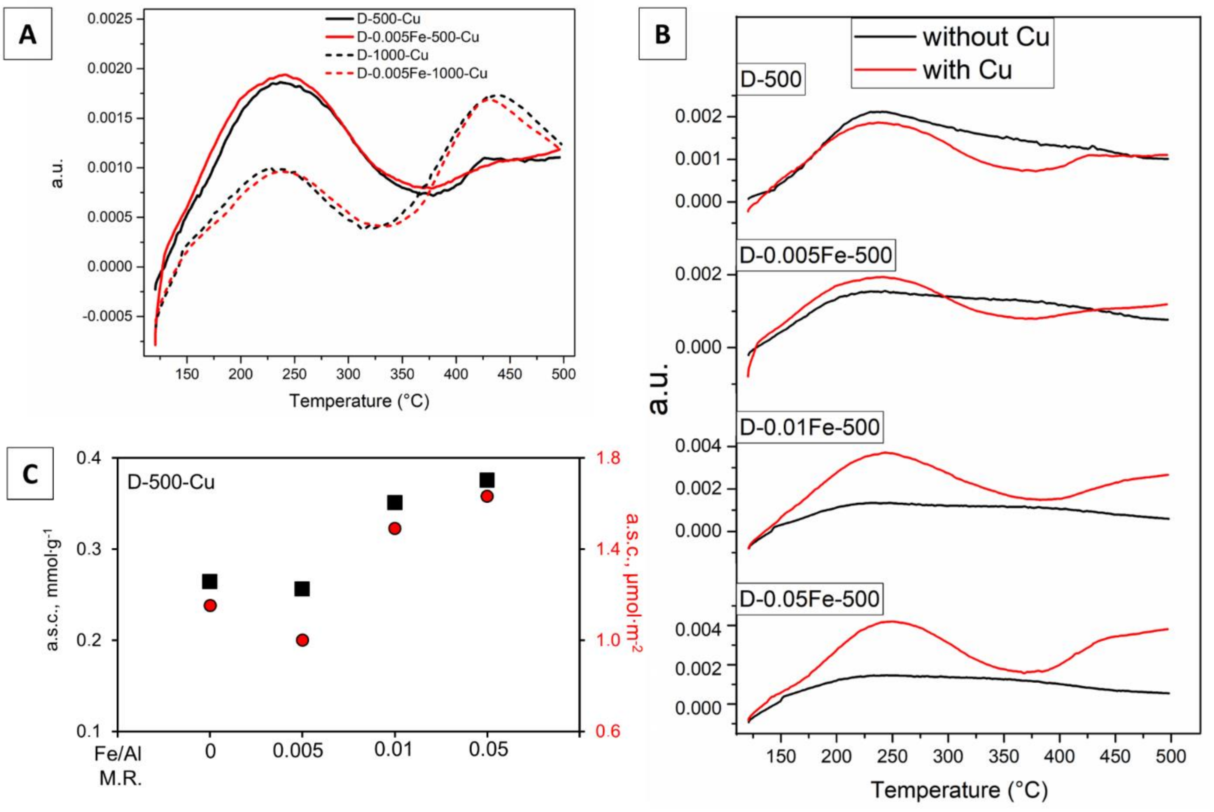

Figure S5. Surface acidity properties of examined samples. (A) Pyridine desorption profiles for catalysts from precursors calcined at 500 and 1000°C, prepared without Fe and with Fe/Al ratio of 0.005. (B) Pyridine desorption profiles for samples with and without copper and with different iron content. (C) Acid site concentration (a.s.c.) per gram (▪) and per m^2 (●) of catalyst, as a function or Fe/Al molar ratio (M.R.) for samples prepared at 500°C.

N_2 physisorption

Figure S6a shows type IV isotherms of the Fe-doped γ-alumina samples with similar hysteresis (H3) shape, indicating the presence of aggregates of plate-like particles with voids consisting of mesopores and macropores. On the other hand, two types of nitrogen isotherm hysteresis can be observed for samples containing iron and copper (Figure S6b), e.g. H3 and H2a. The latter can be found for samples with higher iron amount (Fe/Al molar ratio 0.01 and 0.05). These isotherms are less intense and imply on presence of mainly mesopores. An apparent

macroporous structure (type II isotherm) transforms to a more mesoporous one (type IV isotherm) (REFERENCE https://doi.org/10.1515/pac-2014-1117).

Figure S7 shows the pore size distribution (PSD) of samples with different Fe/Al ratios (Figure S7a), and their evolution during preparation of copper containing catalyst of the same origin (Figure S7b). An evident broad pore size distribution in 0.01 and 0.05 Fe/Al containing samples changed to a structure with more uniform mesopores after copper loading. Copper deposition led to a decrease of larger mesopores and macropores (20-60 nm), for example at higher iron amount only mesopores below 16 nm can be observed. Pore size distribution is also significantly altered after copper introduction in sample D-500, where broad multimodal PSD with maxima at 30 and 44 nm changed to bimodal PSD with less intense maxima. Interestingly, the PSD of sample D-0.005Fe-500-Cu is broader in comparison to other presented materials with Cu and it remains preserved if compared to the material before copper impregnation.

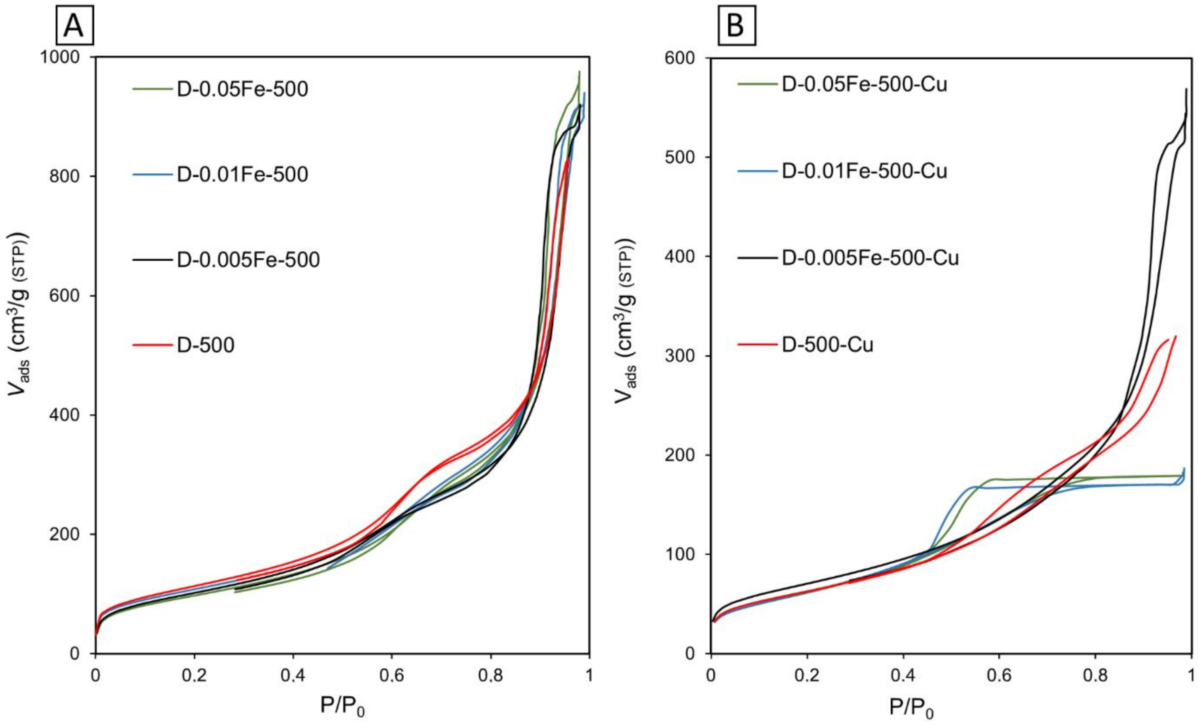

Figure S6. Nitrogen physisorption of samples with different Fe content, calcined at 500°C (A), and impregnated with Cu (B).

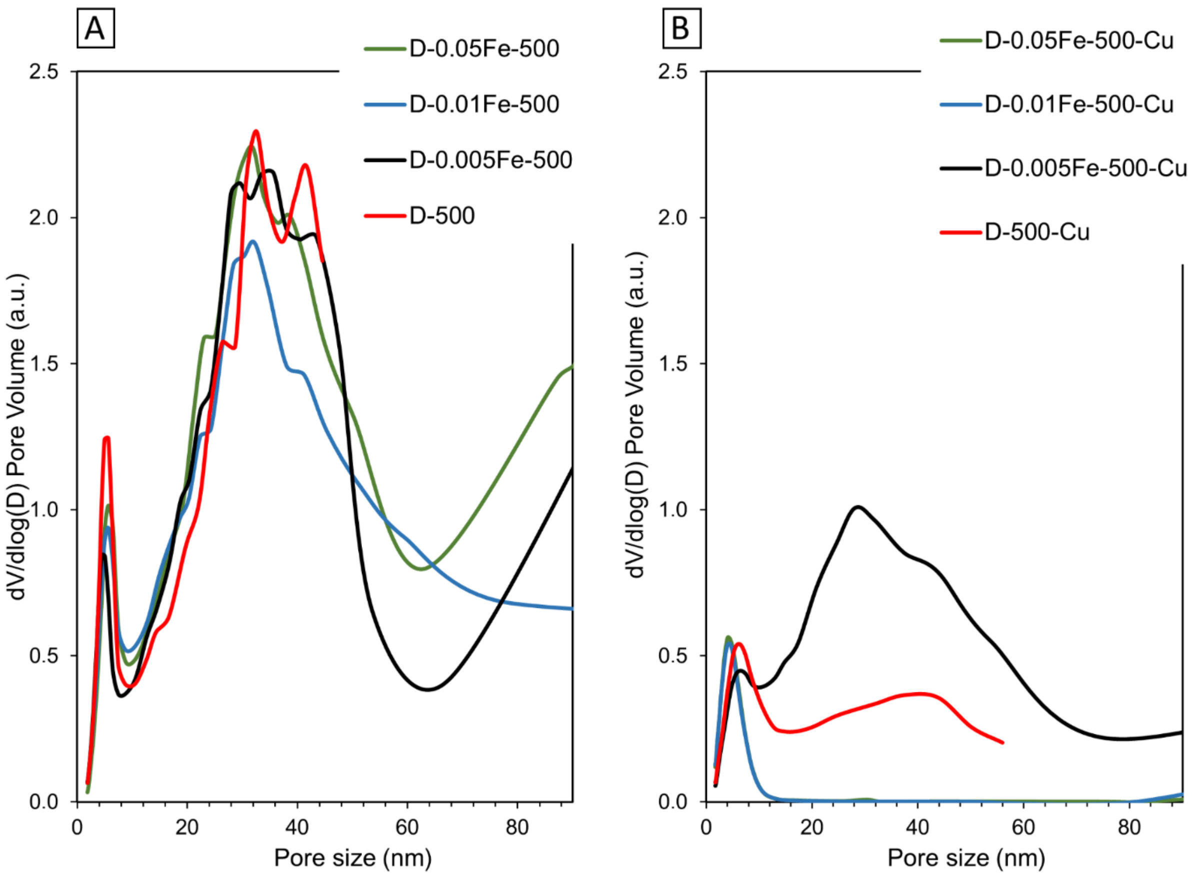

Figure S7. Pore size distribution (PSD) of samples with different iron content, calcined at 500°C (A) and impregnated with copper (B).

NMR

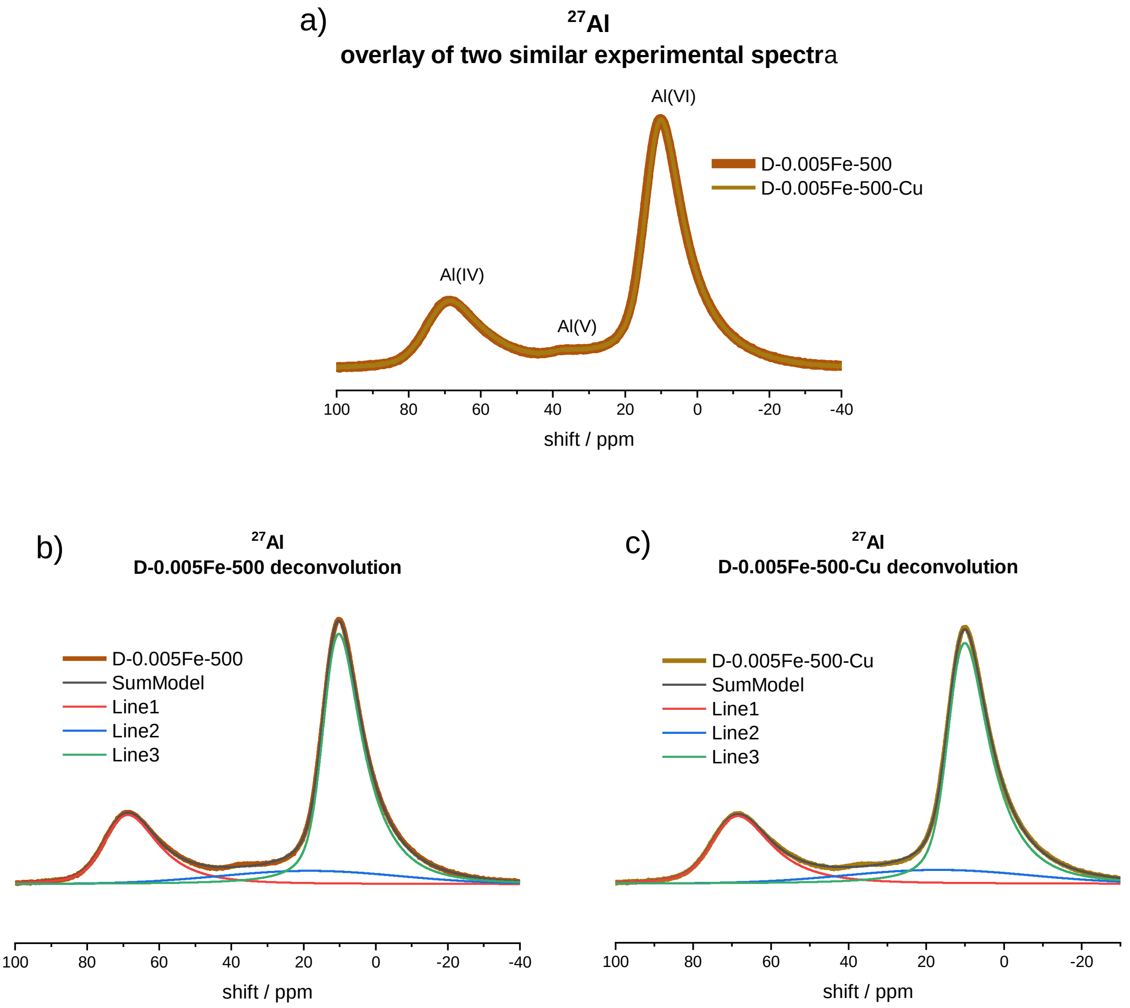

Figure S8. Comparison of ^{27}Al MAS NMR spectra of samples of D-0.005Fe-500 and D-0.005Fe-500-Cu. Not only that the two spectra look almost identical at a quick look , they can also be fitted with an equal model, which comprises three well resolved contributions belonging to four-coordinated aluminum species (isotropic shift of 73 ppm, C_Q of 5.1 MHz, relative intensity of 23 %), five-coordinated aluminum species (isotropic shift of 20 ppm, C_Q of 2.6 MHz, and, and relative intensity of 13 %), and six-coordinated aluminum species (isotropic shift of 14 ppm, C_Q of 4.8 MHz, and relative intensity of 64 %) .The spectra were fitted/modelled with dmfit (REFERENCE https://doi.org/10.1002/mrc.984). Both spectra also exhibit rather broad lines. Therefore all three contributions were modelled by The "CzSimple" 'lineshape', which implements the Czjzek distribution of quadrupolar interaction (REFERENCE https://doi.org/10.1016/j.gca.2004.05.048).

XAS

The catalyst samples were prepared in the form of homogeneous pellets, pressed from micronized sample powder, with the total absorption thickness (μd) of about 2.5 above the investigated Cu or Fe K-edge. Si (111) double crystal monochromator with about 1 eV resolution at 9 keV was used. The intensity of the monochromatic X-ray beam was measured by 30 cm long ionization chambers detectors filled with a N_2, Ar, He gas mixture at 2 bars, such to have 20% of absorption for the I_0 detector, 80% of absorption for the I1 detector and 95% of absorption for the I_2 detector. Sample pellets were placed in the beam between first two ionization chambers detectors. For fluorescence detection, the samples were rotated 45° with respect to the X-ray beam. Fluorescence signal was detected with SDD solid state fluorescence detector. The absorption spectra were measured in the energy region from -150 eV to +1200 eV relative to the Fe or Cu K-edge. In the XANES region equidistant energy steps of 0.3 eV were used, while for the EXAFS region equidistant k steps of 0.03 Å^{-1} were adopted, with an integration time of 2 s/step in transmission mode and 5s/ step in fluorescence mode. Three repetitions of the scans were superimposed to improve the signal-to-noise ratio. The exact energy calibration at Fe and Cu K-edge was established with simultaneous absorption measurement on a 5 μm thick Fe or Cu metal foil, respectively, placed between the second and the third ionization chamber. The first inflection point of the spectrum of the Fe foil at Fe K-edge was assigned at 7112 eV and that of the Cu foil at Cu K-edge was assigned at 8979.0 eV. Absolute energy reproducibility of the measured spectra was ±0.01 eV.

XANES

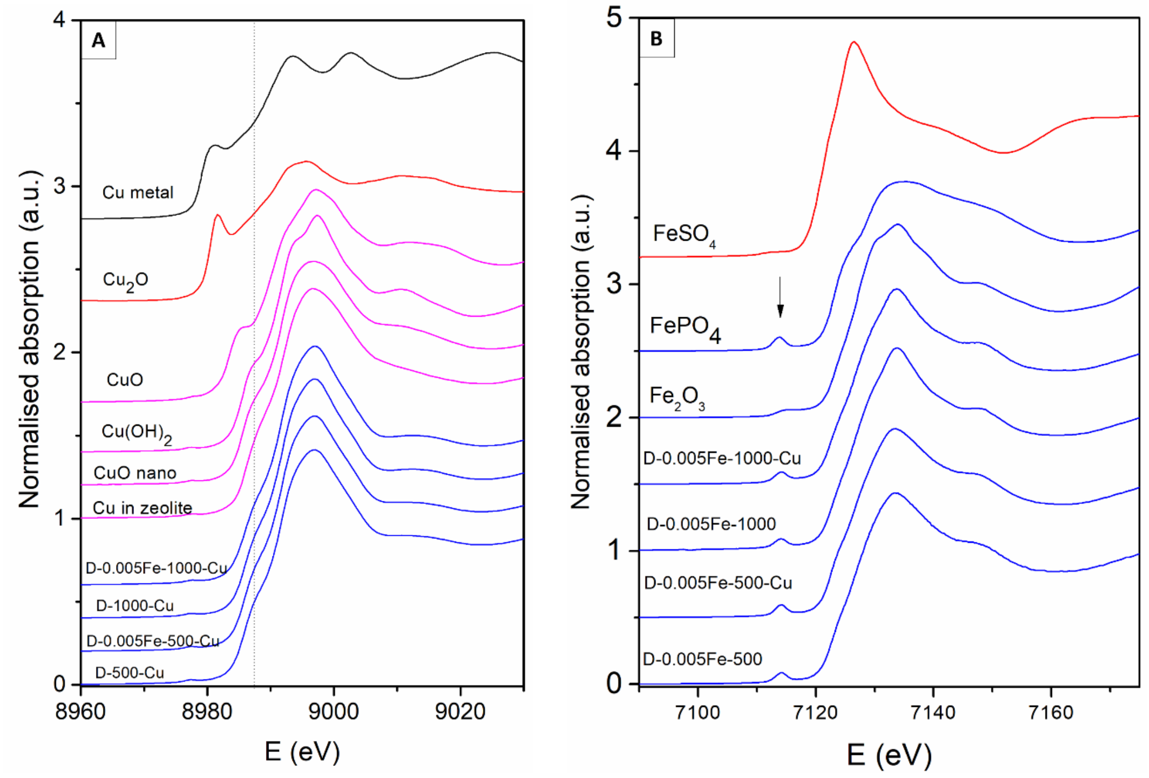

Figure S9. (A) Normalized Cu K-edge XANES spectra measured on Cu and Fe functionalized alumina samples. The spectrum of Cu metal, the spectrum of crystalline Cu_2O as a reference for Cu^{1+}, and the spectra of crystalline CuO, nanoparticles CuO, crystalline Cu-hydroxide and Cu incorporated in zeolite, as a reference for Cu^{2+}, are shown for comparison. Vertical dotted line is plotted at the Cu 1s-4p pre-edge absorption feature (8987eV) in Cu-hydroxide bulk crystal. (B) Normalized Fe K-edge XANES spectra measured on Fe and Cu functionalized alumina samples. The spectrum of crystalline $FeSO_4$ as a reference for Fe^{2+}, and the spectra of crystalline Fe_2O_3 and $FeP0_4$, as a reference for Fe^{3+}, with octahedral and tetrahedral symmetry, respectively, are shown for comparison. Arrow indicates Cu 1s-4p pre-edge resonance (7114 eV) characteristic for tetrahedrally coordinated Fe cations.

Table S1. Parameters of the nearest coordination shells around Cu in Fe and Cu functionalized alumina samples: average number of neighbor atoms (N), distance (R), and Debye-Waller factor (σ^2). Uncertainty of the last digit is given in parentheses. A best fit is obtained with the

amplitude reduction factor S_0^2=0.80 and the shift of the energy origin ΔE_o=-1(1) eV. The R-factor (quality of fit parameter) is listed in the last column.

Neighbours	*N*	*R* [Å]	σ^2 [$Å^2$]	*R*-factor
**D-500-Cu**				
O	4	1.937(6)	0.0060(3)	0.00005
O	2	2.36(2)	0.020(9)	
Al	2.2(7)	2.88(3)	0.015(5)	
Al	1.9(7)	3.14(3)	0.015(5)	

Neighbours	*N*	*R* [Å]	σ^2 [$Å^2$]	*R*-factor
**D-0.005Fe-500-Cu**				
O	4	1.937(2)	0.0060(3)	0.00001
O	2	2.37(2)	0.020(9)	
Al	2.2(7)	2.89(2)	0.015(5)	
Al	1.9(7)	3.16(3)	0.015(5)	

Neighbours	*N*	*R* [Å]	σ^2 [$Å^2$]	*R*-factor
**D-1000-Cu**				
O	4	1.945(2)	0.0056(5)	0.00007
O	2	2.35(2)	0.027(9)	
Al	1.2(7)	2.85(4)	0.013(5)	
Al	1.6(7)	3.13(3)	0.013(5)	

Neighbours	*N*	*R* [Å]	σ^2 [$Å^2$]	*R*-factor
**D-0.005Fe-1000-Cu**				

O	4	1.946(9)	0.0055(4)	0.00006
O	2	2.33(2)	0.030(9)	
Al	1.0(5)	2.84(4)	0.013(5)	
Al	1.7(5)	3.12(5)	0.013(5)	

Table S2. Parameters of the nearest coordination shells around Fe in Fe and Cu functionalized alumina samples: average number of neighbor atoms (N), distance (R), and Debye-Waller factor (σ^2). Uncertainty of the last digit is given in parentheses. A best fit is obtained with the amplitude reduction factor S_0^2=0.80 and the shift of the energy origin ΔE_o=-3(1) eV. The R-factor (quality of fit parameter) is listed in the last column.

Neighbours	*N*	*R* [Å]	σ^2 [Å^2]	*R*-factor
**D-0.005Fe-500**				
O	5.3(7)	1.94(1)	0.008(1)	0.00014
O	0.6(4)	2.49(1)	0.005(1)	
Fe	0.7(2)	3.04(3)	0.004(2)	
Al	1.4(5)	3.46(2)	0.005(2)	
Fe	1.3(3)	3.84(3)	0.004(2)	

Neighbours	*N*	*R* [Å]	σ^2 [Å^2]	*R*-factor
**D-0.005Fe-500-Cu**				
O	4.6(7)	1.91(1)	0.007(1)	0.00019
O	0.9(4)	2.47(1)	0.004(1)	
Fe	0.6(2)	3.03(3)	0.004(2)	
Al	2.4(5)	3.37(2)	0.005(2)	
Fe	0.6(3)	3.69(3)	0.004(2)	

Neighbours	*N*	*R* [Å]	σ^2 [Å^2]	*R*-factor
**D-0.005Fe-1000**				
O	5.1(9)	1.94(1)	0.008(2)	0.00014
O	0.8(4)	2.51(1)	0.005(2)	
Fe	0.8(2)	3.05(3)	0.004(2)	
Al	1.2(5)	3.23(2)	0.005(2)	
Fe	0.6(3)	3.48(3)	0.004(2)	

Neighbours	*N*	*R* [Å]	σ^2 [Å^2]	*R*-factor
**D-0.005Fe-1000-Cu**				
O	4.1(5)	1.93(1)	0.007()	0.00011
O	1.6(4)	2.51(2)	0.005(1)	
Fe	0.6(2)	3.08(3)	0.004(2)	
Al	2.5(5)	3.32(2)	0.005(2)	
Fe	0.7(3)	3.58(3)	0.004(2)	

XRD analysis of repeated heating and cooling of catalyst

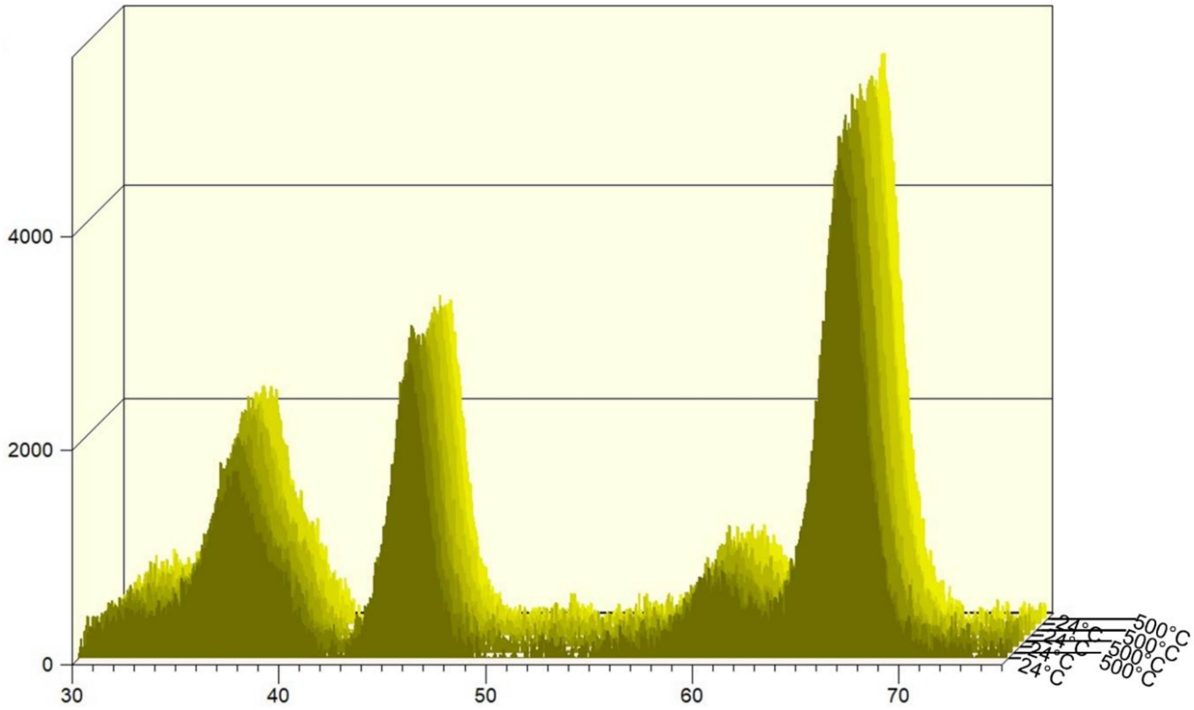

Figure S10. In-situ XRD analysis of D-0.005Fe-500-Cu catalyst during repeated heating and cooling from the room temperature to 500°C. The structure of the material remained intact as no additional diffraction lines, or lack of them, were observed with this analysis for four repeated cycles.

Pulsed toluene oxidation reaction

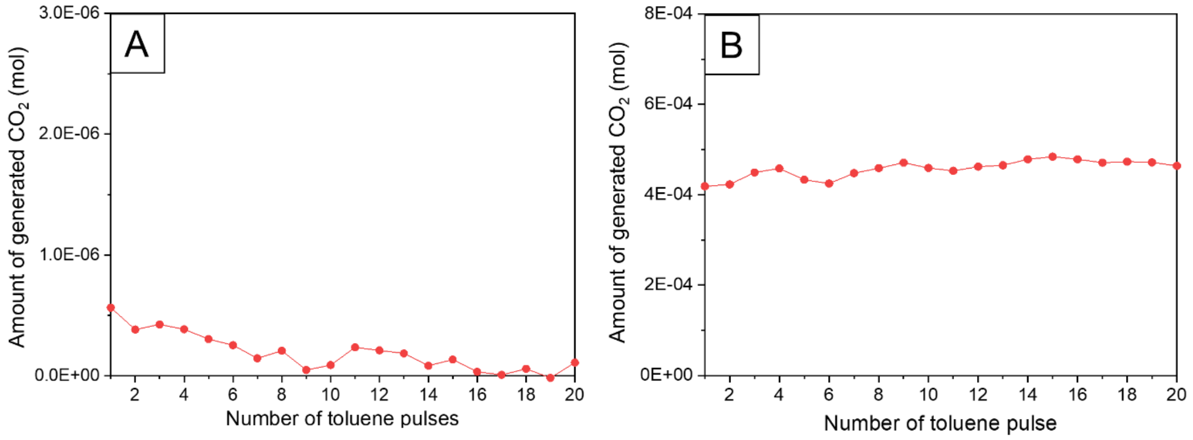

Figure S11. Amount of generated CO_2 as a function of toluene pulses obtained at T=380°C in the presence of D-500-Cu catalyst in a) inert and b) air atmosphere.

CHN elemental analysis

CHN elemental analysis was performed on spent samples D-500-Cu, D-1000-Cu and D-0.005Fe-1000-Cu to determine the potential coke formation and differences in catalytic activity among those samples. Table S3 shows that the spent samples D-500-Cu and D-0.005Fe-500-Cu contain 0.6 and 0.5 wt.% carbon, respectively. This indicates the formation of some organic species as side products, which remain adsorbed on the catalyst surface. Usually, coke formation leads to reduced activity after certain TOS. A significantly higher carbon deposition was observed on sample D-1000-Cu in comparison to other two samples, prepared at 500°C.

Table S3. Carbon content (wt.%) in spent samples, determined by means of CHN analysis.

**Sample after catalytic run**	**Carbon content, wt.%**
**D-500-Cu**	0.6
**D-1000-Cu**	0.9
**D-0.005Fe-500-Cu**	0.5

Band gap analysis

Figure. S13 displays the band gap energy for alumina sample (D-500), cupper-alumina sample (D-500-Cu), iron-alumina-samples (D-0.005Fe-500, D-0.01Fe-500, D-0.05Fe-500) and cupper-iron-alumina samples (D-0.005Fe-500-Cu, D-0.01Fe-500-Cu, D-0.05Fe-500-Cu). Interesting observation was made when comparing the iron-alumina samples without cupper with iron-alumina samples with cupper. In all cases, except for the material D-0.005Fe-500-Cu, the band gap energy was reduced after introduction of Cu to the alumina or iron--alumina support. This reduction was intensified with increased Fe/Al ratio. In case of the sample with Fe/Al ration of 0.005, this was although not the case, and the band gap energy was increased. The results show that the part of iron is embedded in the Al_2O_3 crystal lattice at Al sites and thus creates additional electronic states in the Al_2O_3 crystal structure within the energy gap below the conduction band. These electronic states extend over the entire crystal surface and act on Cu, which is bound to the Al_2O_3 surface. Cu binds to the surface after Fe is already incorporated into Al_2O_3.

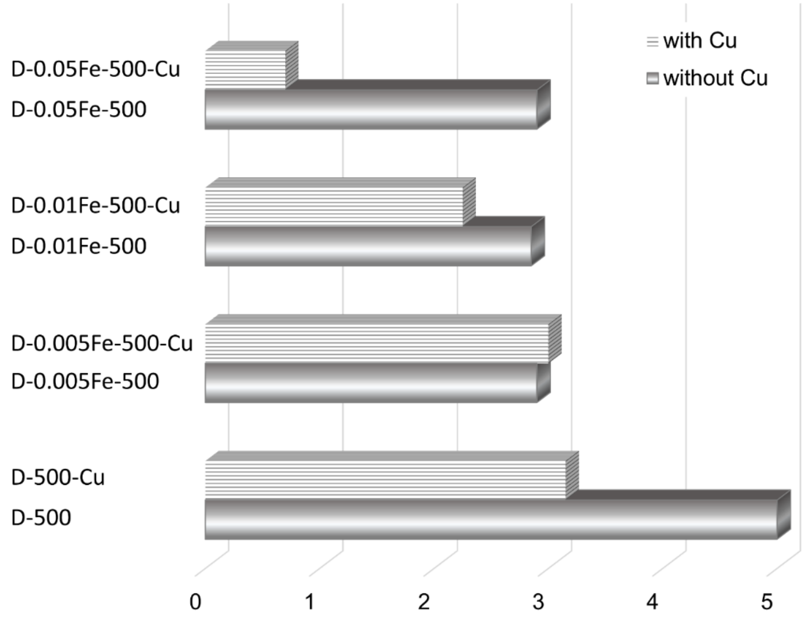

Figure S12. Comparison of bandgap energies supporting electronic interactions between CuO_x clusters and iron-alumina-s
